# Modeling climate-driven changes in potential zoonotic virus density across Australia

**DOI:** 10.1016/j.isci.2026.117414

**Published:** 2026-09-01

**Authors:** Maryam Golchin, Justin D. Sexton, Roslyn I. Hickson

**Affiliations:** 1 Commonwealth Scientific and Industrial Research Organisation (CSIRO), Townsville, QLD 4811, Australia; 2 Australian Institute of Marine Science (AIMS), Cape Cleveland, QLD 4810, Australia; 3 Australian Institute of Tropical Health and Medicine, James Cook University, Townsville, QLD 4811, Australia

**Keywords:** climate change, zoonotic viruses, potential zoonotic virus density, spillover risk, machine learning, Australia

## Abstract

Over 70% of emerging infectious diseases originate from zoonotic sources, making spillover risk prediction important for public health preparedness. This study investigates how potential zoonotic virus density (PVD) may change across Australia in 2050 under climate change. PVD captures ecological hazard, while actual spillover risk additionally depends on wildlife-human contact and pathogen transmission dynamics. Using an Extra Trees Regressor trained on 2020 climate outputs from eight global climate models (GCMs) under two emissions scenarios, and using projected 2020 PVD as the response variable, we generated spatial re-projections of climate suitability for PVD under 2050 climate conditions. Re-projections revealed spatial redistribution rather than a uniform change, with higher projected suitability across parts of western and southwestern Australia and lower suitability in some eastern areas relative to the 2020 projections. Relative humidity was the strongest overall climatic predictor, while the SHapley Additive exPlanations (SHAP) analysis highlighted model-specific effects of precipitation, water vapor, and solar radiation. Local Moran’s I showed persistent spatial clustering across coastal and inland areas.

## Highlights

• Projected climate change spatially redistributes zoonotic virus density in Australia
• Relative humidity is the strongest predictor of climate-driven virus density
• Projected suitability increases in western, decreases in eastern coastal Australia
• Spatial clustering of projected zoonotic virus density persists under warming

## Graphical abstract

**Figure:**
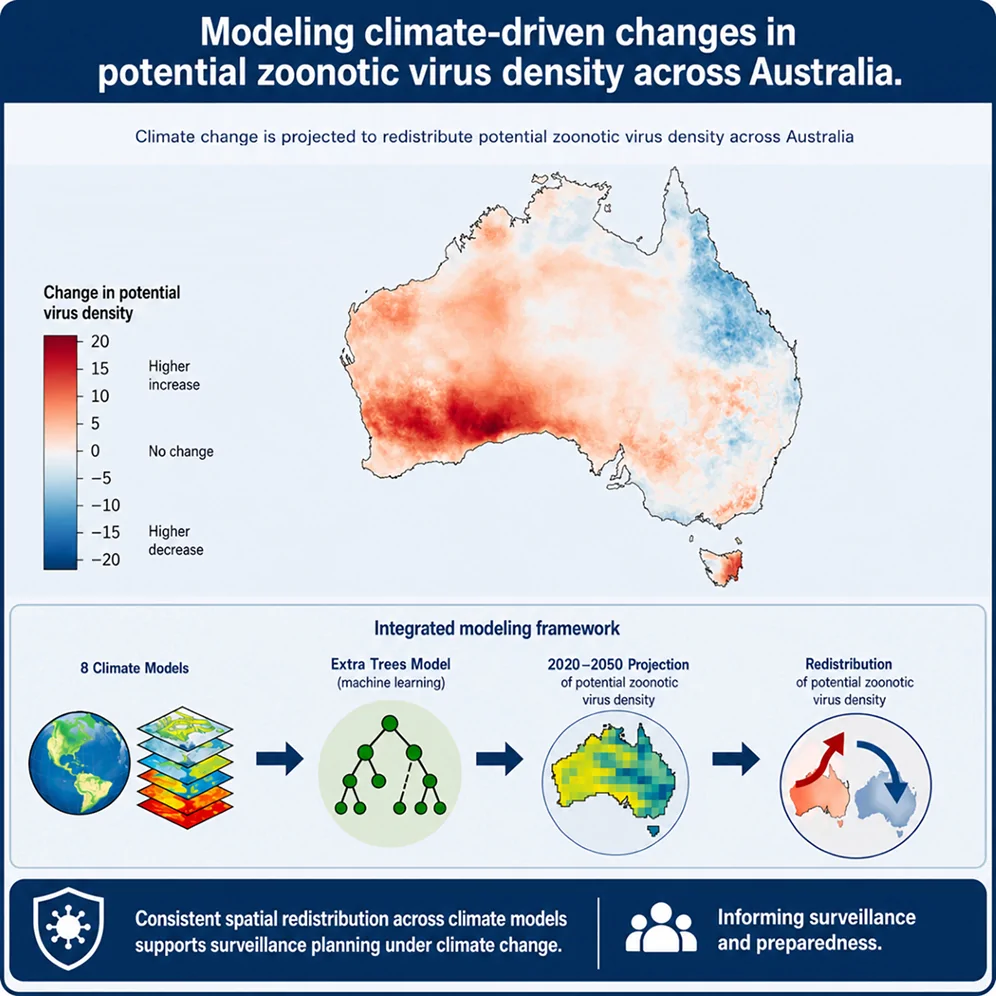

## Teaser

Public health; Environmental science; Environmental health; Global change; Virology

## Introduction

Climate change plays an important role in shaping ecological and biological processes, with far-reaching implications for public health, particularly through its impact on zoonotic disease transmission. One critical concern is how climate change influences zoonotic spillover risk, the transmission of pathogens from animals to humans. Recent outbreaks, including COVID-19, underscore the severity of zoonotic spillover as a threat to global health security. Several studies have established a correlation between climate variables and zoonotic pathogen emergence,^1^^,^^2^^,^^3^ highlighting the need to mitigate spillover risks in a warming world.

Research demonstrates that climate change can alter the habitats and behaviors of wildlife and humans, creating new pathways for pathogen transmission.^1^^,^^4^ Changes in temperature, precipitation patterns, and extreme weather events can alter the distribution and abundance of reservoir hosts and vectors, increasing or decreasing the likelihood of spillover events. Furthermore, climate-induced land-use changes and human activities intensify human-wildlife interactions, amplifying or reducing zoonotic disease risks.

Changes in temperature and precipitation patterns can reduce species’ climatic suitability, forcing wildlife to migrate to more favorable habitats.^5^ These range movements may increase the likelihood of novel interactions between species, which may lead to pathogen spillover as hosts encounter new environments and reservoirs. Species with narrow climatic tolerances or limited dispersal abilities face heightened risks of extinction, reducing biodiversity, and further destabilizing ecosystems.^5^ Such disruptions can lead to the loss of natural checks and balances within ecosystems, potentially favoring the increase of reservoir hosts and vectors associated with zoonotic pathogens.

Human activities also have substantial impacts. Land use changes, such as deforestation and agricultural expansion, have long been associated with emerging infectious diseases.^6^ These human-driven changes fragment habitats, shift species distributions, and increase contact between wildlife and humans, facilitating pathogen transmission. Taylor et al.^6^ emphasize that habitat fragmentation not only exposes humans to novel pathogens but also creates ecological conditions contributing to spillover by concentrating wildlife populations into smaller, high-contact areas. While land use change, wildlife trade, and agricultural intensification are recognized drivers of zoonotic spillover, their mechanistic pathways are difficult to represent across Australia due to data limitations. In contrast, climate variables can be derived from validated reanalysis products and projected through CMIP5 ensembles, making them suitable for quantitative modeling. The present study therefore focuses specifically on climate as a driver of ecological hazard, while acknowledging that human activities influence exposure and transmission and should be incorporated into future modeling frameworks.

Zoonotic viruses have been linked to habitat loss, agricultural change, and shifts in wildlife populations.^7^ These factors, often intensified by climate change, interact in complex ways and raise concerns about increased spillover risk, particularly in areas with high viral density. Predicting zoonotic spillover risk remains challenging because it is shaped by many interacting drivers, including biodiversity loss, land use change, and altered pathogen-host dynamics.^8^ Viral density can be used as a proxy for zoonotic spillover risk,^9^ although it remains uncertain whether areas with high viral density consistently correspond to higher spillover risk or whether this relationship is more context-dependent.

In Australia, understanding the relationship between climate variables and spillover risk is particularly important given the continent’s unique biodiversity and vulnerability to climate change.^10^ In this study, we model the relationship between projected climate variables and estimated potential zoonotic virus density as a proxy for spillover risk across Australia. Potential zoonotic virus density, as developed by Golchin et al.,^9^ reflects the ecological capacity of a landscape to support zoonotic viruses associated with mammalian and avian hosts, given current species distributions. It represents an upstream component of spillover risk, capturing ecological hazard rather than human disease incidence, which also depends on wildlife-human contact, susceptible human populations, and pathogen-specific transmission dynamics.

To project future climate conditions, we used outputs from eight global climate models (GCMs):

• MIROC5^11^: Model for Interdisciplinary Research on Climate, version 5.
• HadGEM2-CC^12^: Hadley Center Global Environment Model, version 2, Carbon Cycle.
• GFDL-ESM2M^13^: Geophysical Fluid Dynamics Laboratory Earth System Model 2M.
• CanESM2^14^: Canadian Earth System Model, version 2.
• CNRM-CM5^15^: Center National de Recherches Météorologiques Climate Model, version 5.
• CESM1-CAM5^16^: Community Earth System Model 1, with the Community Atmosphere Model, version 5.
• NorESM1-M^17^: Norwegian Earth System Model, version 1, medium resolution.
• ACCESS1-0^18^: Australian Community Climate and Earth-System Simulator, version 1.0.

Using multiple GCMs allowed us to capture variation among climate model projections and assess how sensitive projected climate suitability for potential zoonotic virus density is to different future climate conditions.

Climate projections were evaluated under two representative concentration pathways (RCPs). RCP 4.5^19^ represents a stabilization scenario in which radiative forcing stabilizes at approximately 4.5 W/m^2^ by 2100, while RCP 8.5^20^ represents a high-emissions scenario in which radiative forcing reaches approximately 8.5 W/m^2^ by 2100. These scenarios allowed us to assess projected climatic suitability for potential zoonotic virus density under moderate mitigation and high emissions futures, respectively.

An Extra Trees Regressor (ETR) was trained to model the relationship between climate variables and potential zoonotic virus density. Spatially structured validation and checkerboard train-test splitting were used to reduce spatial leakage and account for spatial autocorrelation during model evaluation. Re-projected density surfaces were then assessed using global and local Moran’s I statistics to quantify overall spatial clustering and identify localized high-density clusters, low-density clusters, and spatial outliers across Australia. ETR feature importance and SHapley Additive exPlanations (SHAP) were used to interpret the climatic drivers of projected density, with SHAP providing additional insight into the direction and magnitude of each variable’s contribution to individual predictions. This study does not attempt to predict actual spillover risk. Rather, it predicts climate-driven changes in the ecological potential for zoonotic virus presence, without accounting for human exposure, host contact rates, pathogen-specific transmission, or social and behavioral factors.

## Results

### Extra Trees Regressor selected as the optimal predictive model

An exploratory model comparison was conducted using a random 70/30 train-test split and climate predictors derived from the ACCESS1-0 model under RCP 4.5. Among the candidate algorithms evaluated with the LazyRegressor framework (Table 1), ETR showed the best performance. It achieved the highest predictive accuracy (coefficient of determination [R^2^] = 0.996; adjusted R^2^ = 0.996) and the lowest prediction error (root mean square error [RMSE] = 0.82). Other ensemble methods, including random forest, bagging, gradient boosting, and AdaBoost, showed comparatively lower performance. Simpler models, such as decision trees, resulted in reduced accuracy despite shorter computation times. These metrics reflect the exploratory model selection step using a random split and a single GCM. Spatial block cross-validation performance for the selected ETR across all GCM × RCP combinations is reported in the following section.

**Table 1:** Model selection

Model	Adjusted R^2^	R^2^	RMSE	Time taken (s)
ExtraTreesRegressor	**0.996**	**0.996**	**0.817**	41.576
RandomForestRegressor	0.993	0.993	1.064	209.694
BaggingRegressor	0.991	0.991	1.209	20.934
DecisionTreeRegressor	0.980	0.980	1.826	**3.266**
GradientBoostingRegressor	0.861	0.861	4.790	60.930
AdaBoostRegressor	0.699	0.699	7.055	12.435

Model selection summary comparing candidate regression algorithms using a random 70/30 train-test split implemented with the “LazyRegressor” class in the “LazyPredict” package. Performance was evaluated using adjusted R^2^, R^2^, RMSE, and computational runtime. Bold values indicate the best performance for each metric.

### Model performance is robust across GCMs and emissions pathways

**Figure 1:**
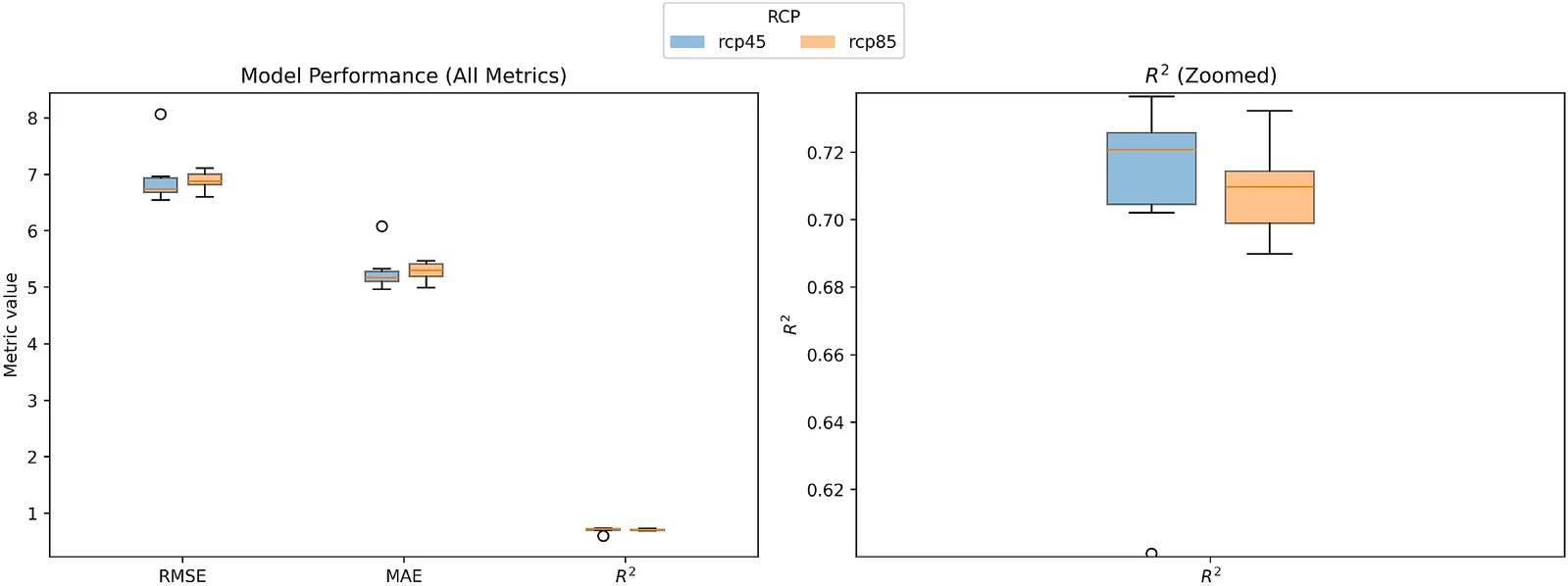
Spatial cross-validation performance of the final Extra Trees Regressor model Distribution of model performance metrics (RMSE, MAE, and R^2^) under checkerboard spatial block cross-validation, aggregated across all eight global climate models for RCP 4.5 (blue) and RCP 8.5 (orange). The right image shows a zoomed view of R^2^ values to highlight inter-scenario variability. See also Figure S2 for individual model performance.

Across all eight GCMs and both RCP scenarios, ETR maintained moderate to high predictive performance on the held-out test set under checkerboard spatial block cross-validation (Figure 1). Across the three test set performance metrics, interquartile ranges are narrow, indicating limited variation among GCM × RCP combinations. R^2^ values are tightly clustered around ∼0.70–0.73 for RCP 4.5 and are slightly lower for RCP 8.5, generally around ∼0.69–0.71, indicating only minor sensitivity to emission pathway. RMSE generally ranges between ∼6.7 and 6.9 and mean absolute error (MAE) generally ranges between ∼5.1 and 5.3. One notable exception is GFDL-ESM2M under RCP 4.5, which does not follow the broader pattern and shows reduced predictive performance. Full model-by-model performance metrics for each GCM × RCP combination are provided in Figure S2.

### Re-projections reveal consistent climate-driven spatial shifts

**Figure 2:**
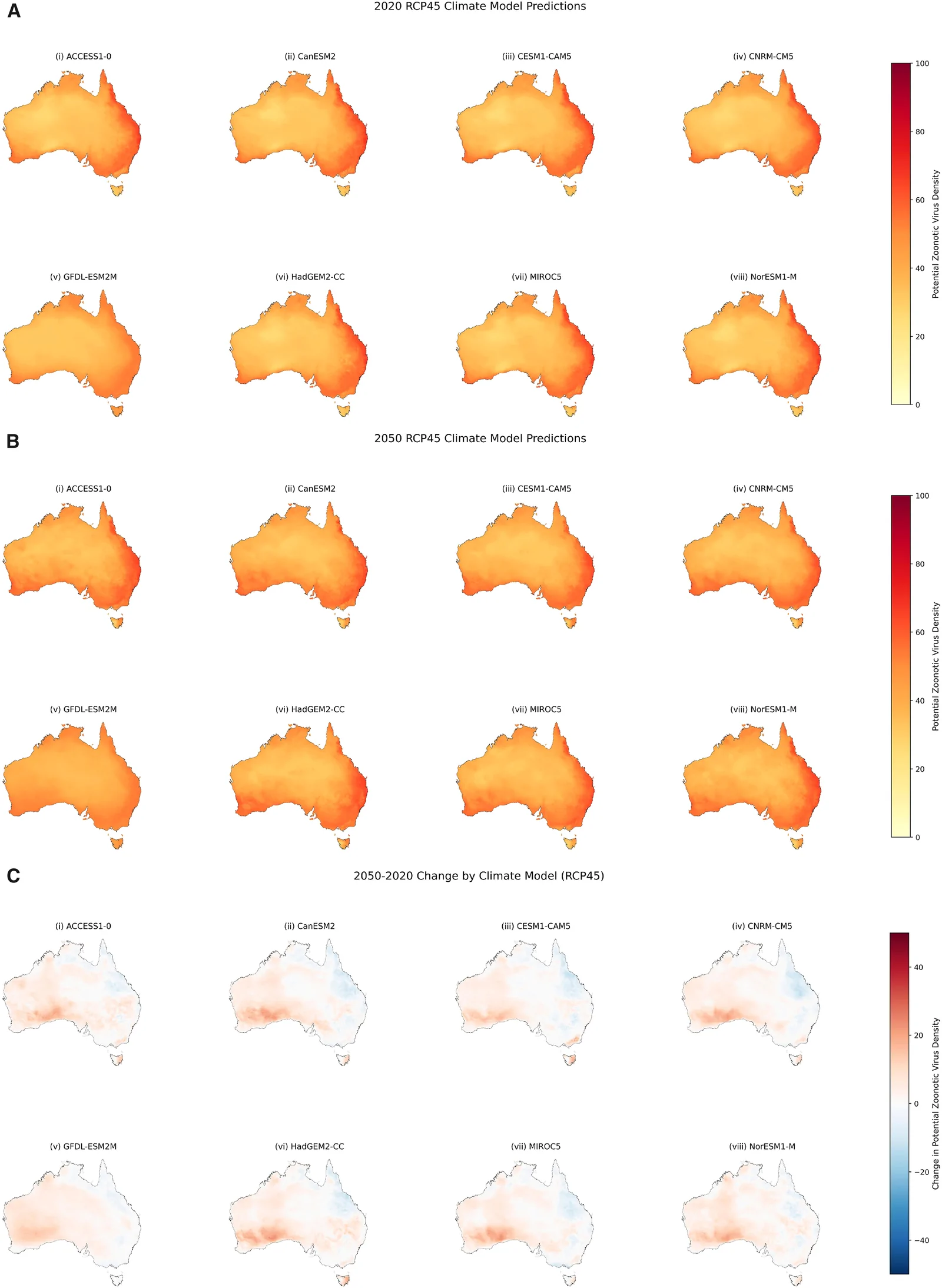
Climate-driven projections of potential zoonotic virus density across Australia under RCP 4.5 (A and B) Projected climate suitability for potential zoonotic virus density for 2020 (A) and 2050 (B) under RCP 4.5, shown for each of the eight global climate models (color scale [0–100]). (C) The change in projected climatic suitability between 2050 and 2020 for each climate model (color scale [−50 to 50]). Red values indicate an increase, and blue values indicate a decrease relative to the 2020 predictions.

**Figure 3:**
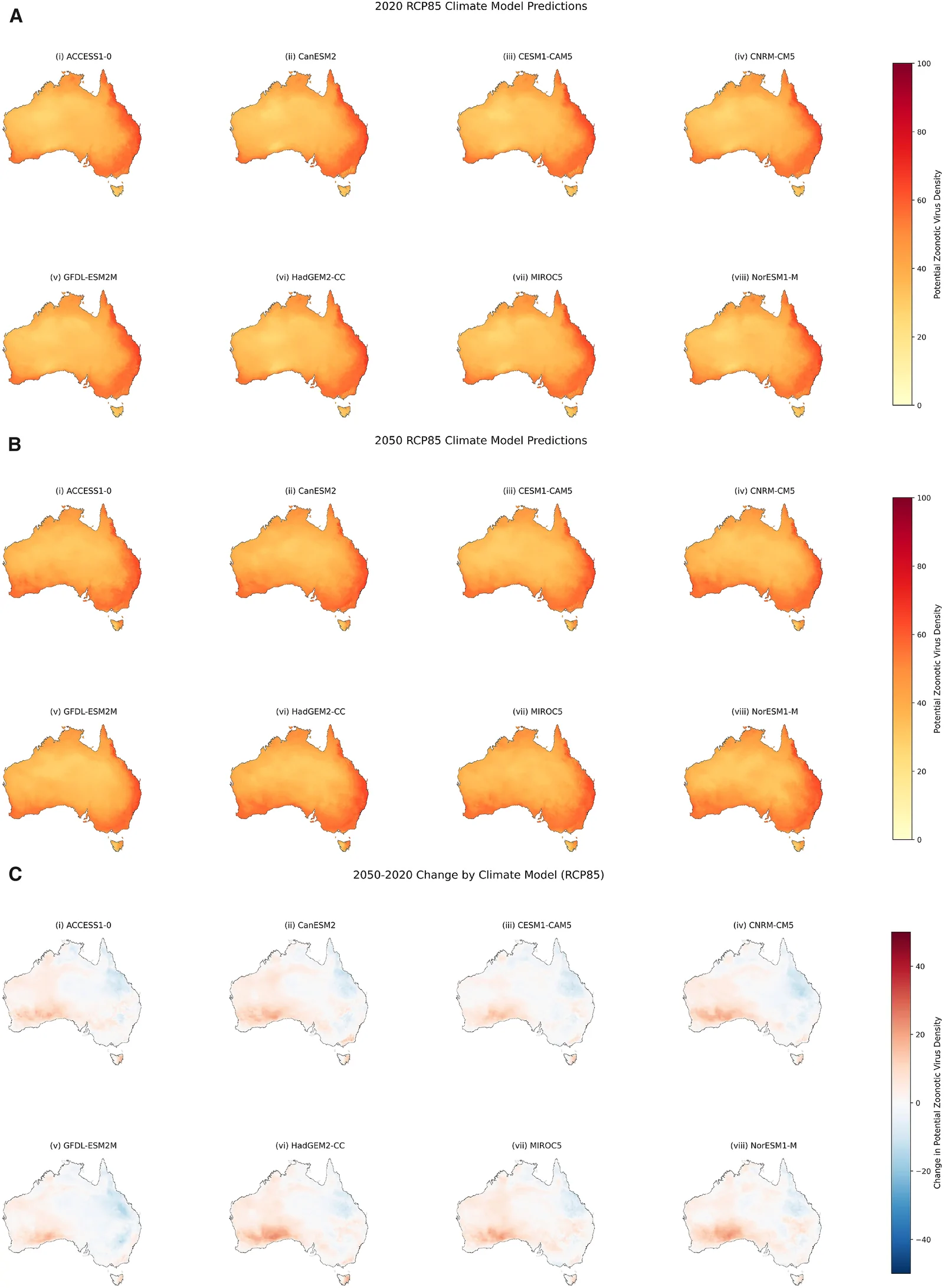
Climate-driven projections of potential zoonotic virus density across Australia under RCP 8.5 (A and B) Projected climate suitability for potential zoonotic virus density for 2020 (A) and 2050 (B) under RCP 8.5, shown for each of the eight global climate models (color scale [0–100]). (C) The change in projected climatic suitability between 2050 and 2020 for each climate model (color scale [−50 to 50]). Red values indicate an increase, and blue values indicate a decrease relative to the 2020 predictions.

Spatial projections for 2020 show a higher potential zoonotic virus density along eastern and southeastern coastal regions and lower values across central and western Australia (Figures 2A, 3A, S3 and S4). By 2050, this pattern shifts (Figures 2B and 3B), with increases across western, central, and parts of southern and northern Australia, and relative decreases or stabilization along portions of the eastern coastal regions.

**Figure 4:**
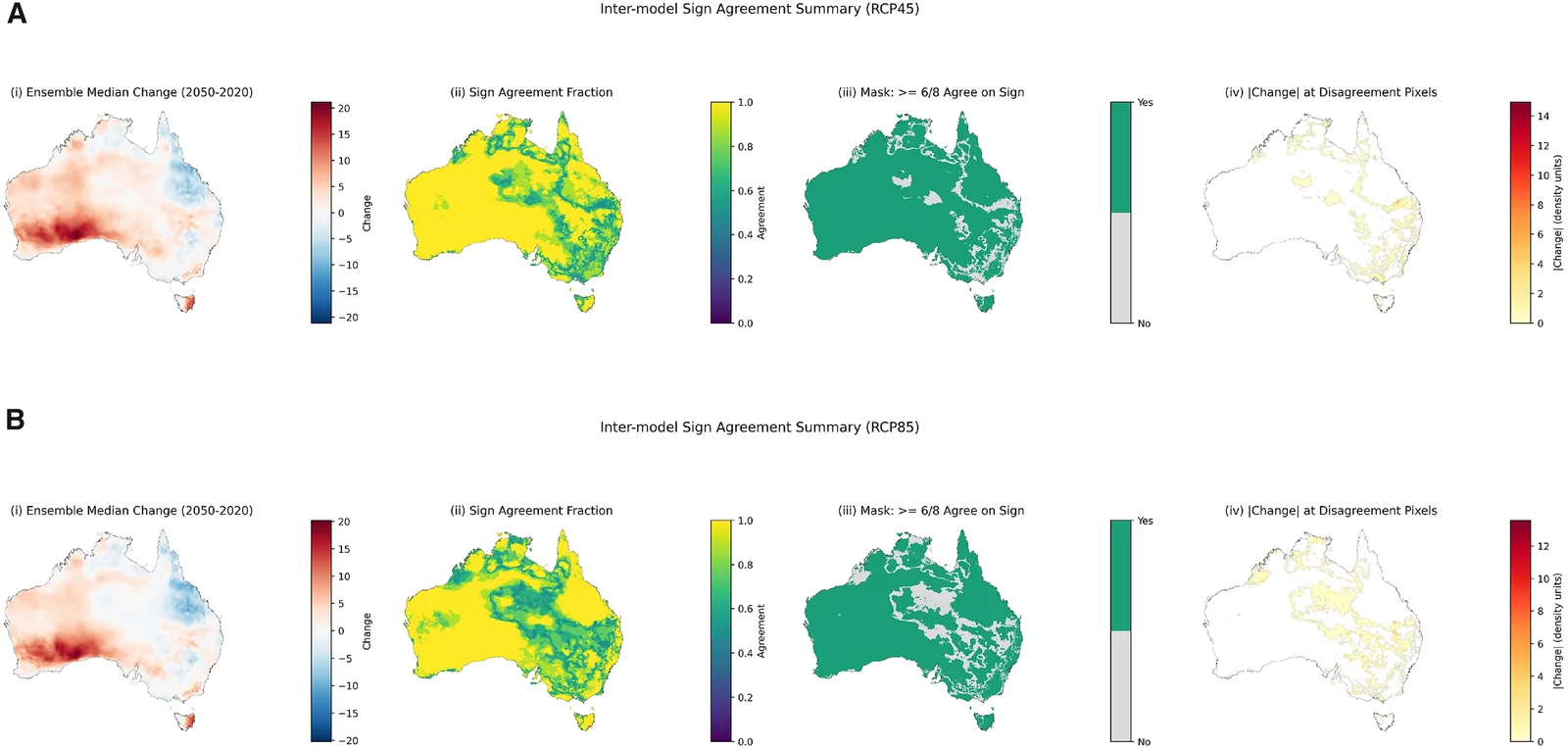
Inter-model agreement in projected changes in potential zoonotic virus density across Australia Inter-model agreement summaries for RCP 4.5 (A) and RCP 8.5 (B). For each scenario, image (i) shows the ensemble median change (2050–2020), image (ii) shows the fraction of climate models agreeing on the sign of change, image (iii) identifies grid cells where at least 6 of 8 models agree on the direction of change, and image (iv) shows the absolute change where less than 6 models agree on the direction of change.

Difference maps (2050–2020) confirm these patterns across all GCMs (Figures 2C and 3C). Positive changes dominate inland and southwestern regions, while negative changes are concentrated along eastern coastal regions. Ensemble median maps (Figures 4A and 4B, image (i)) show strong increases in southwestern Australia and moderate declines in the northeast.

Inter-model agreement is high (Figures 4A and 4B, images (ii and iii)): large areas of Australia show agreement among six or more of the eight GCMs on the direction of change, particularly across western regions, indicating robust spatial signals despite differences in model magnitude. Disagreement is spatially limited, mainly to regions where changes in density are small (Figures 4A and 4B, image (iv)).

The 2020 validation residual maps (Figures S3 and S4) show broadly similar spatial patterns across GCMs and RCP scenarios. Relative residuals, calculated as (*predicted* *values* - *baseline* *values*) / *baseline* *values*, are distributed across Australia rather than concentrated in a single region. Across models, approximately 45%–49% of grid cells show positive residuals, indicating overprediction, and approximately 51%–55% show negative residuals, indicating underprediction. Mean relative residuals are small and positive, generally ranging from approximately 0.015 to 0.021, indicating that predictions are on average around 1.5%–2.1% higher than baseline values. Median relative residuals are slightly negative, ranging from approximately −0.006 to −0.017, suggesting that more than half of grid cells show small underprediction relative to the baseline values.

### Spatial redistribution indicates homogenization under RCP 8.5

**Figure 5:**
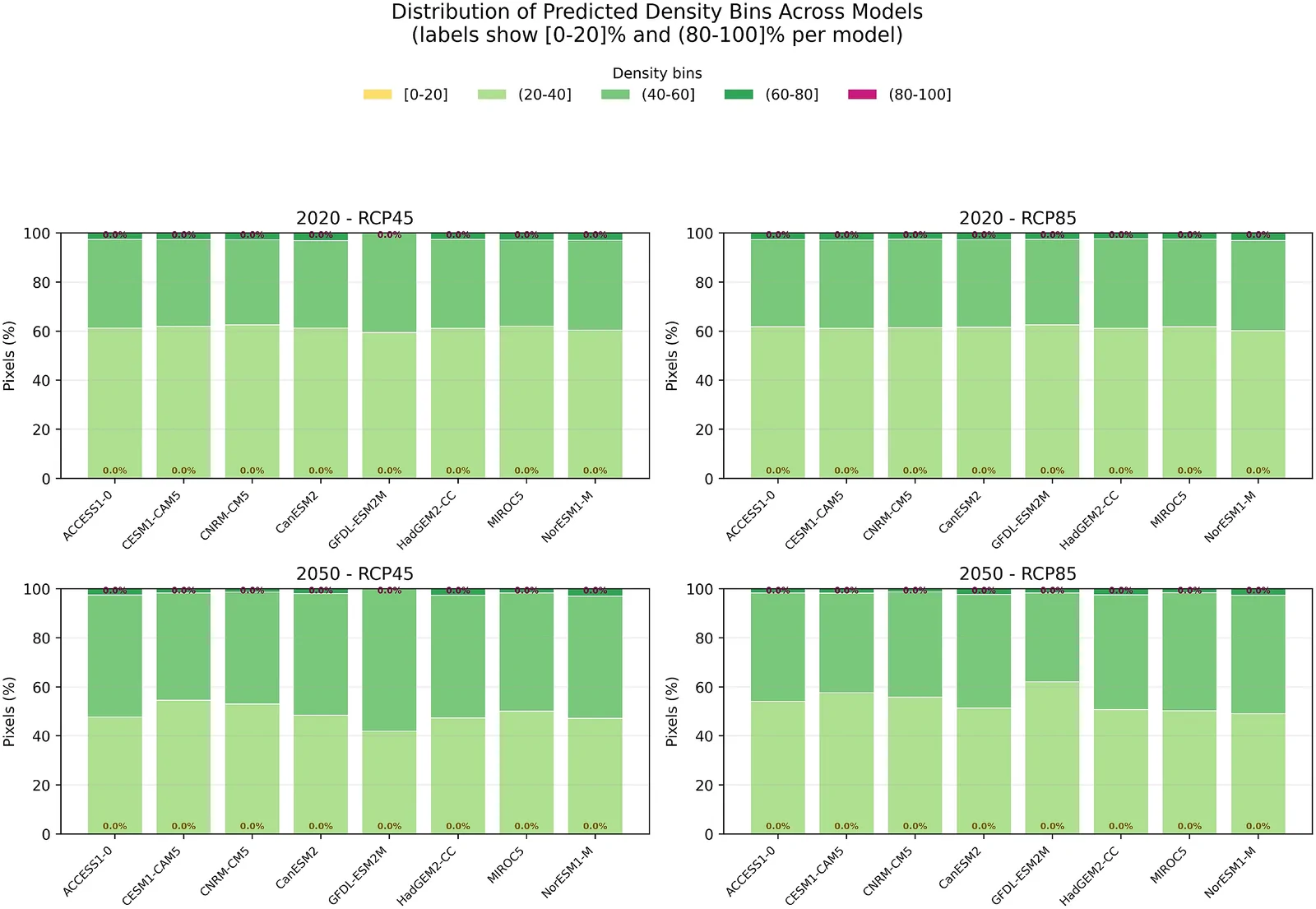
Redistribution of projected climate suitability for potential zoonotic virus density across climate models Stacked bar charts showing the proportional distribution of grid cells across potential zoonotic virus density bins for each global climate model in 2020 (top row) and 2050 (bottom row) under RCP 4.5 (left column) and RCP 8.5 (right column). Values annotated on bars highlight the lowest [0–20] and highest [80–100] density classes. See also Table S2 for detailed values across climate models and emission scenarios for the years 2020 and 2050.

The density bin analysis (Figure 5) showed a shift in the distribution of predicted potential zoonotic virus density between 2020 and 2050. Across the GCMs, most grid cells were concentrated in the 20–80 density classes, particularly the 20–40 and 40–60 density bins. Very low and very high-density bins were not observed.

By 2050, the proportion of grid cells in the 40–60 bin generally increased, while the proportion in the 20–40 bin declined in all models except for one model under the RCP 8.5 scenario. This indicates a shift toward intermediate density values rather than an expansion of very high-density areas. The 60–80 bin remained relatively small across models and scenarios.

### Uncertainty analyses highlight spatial consistency with localized variability

**Figure 6:**
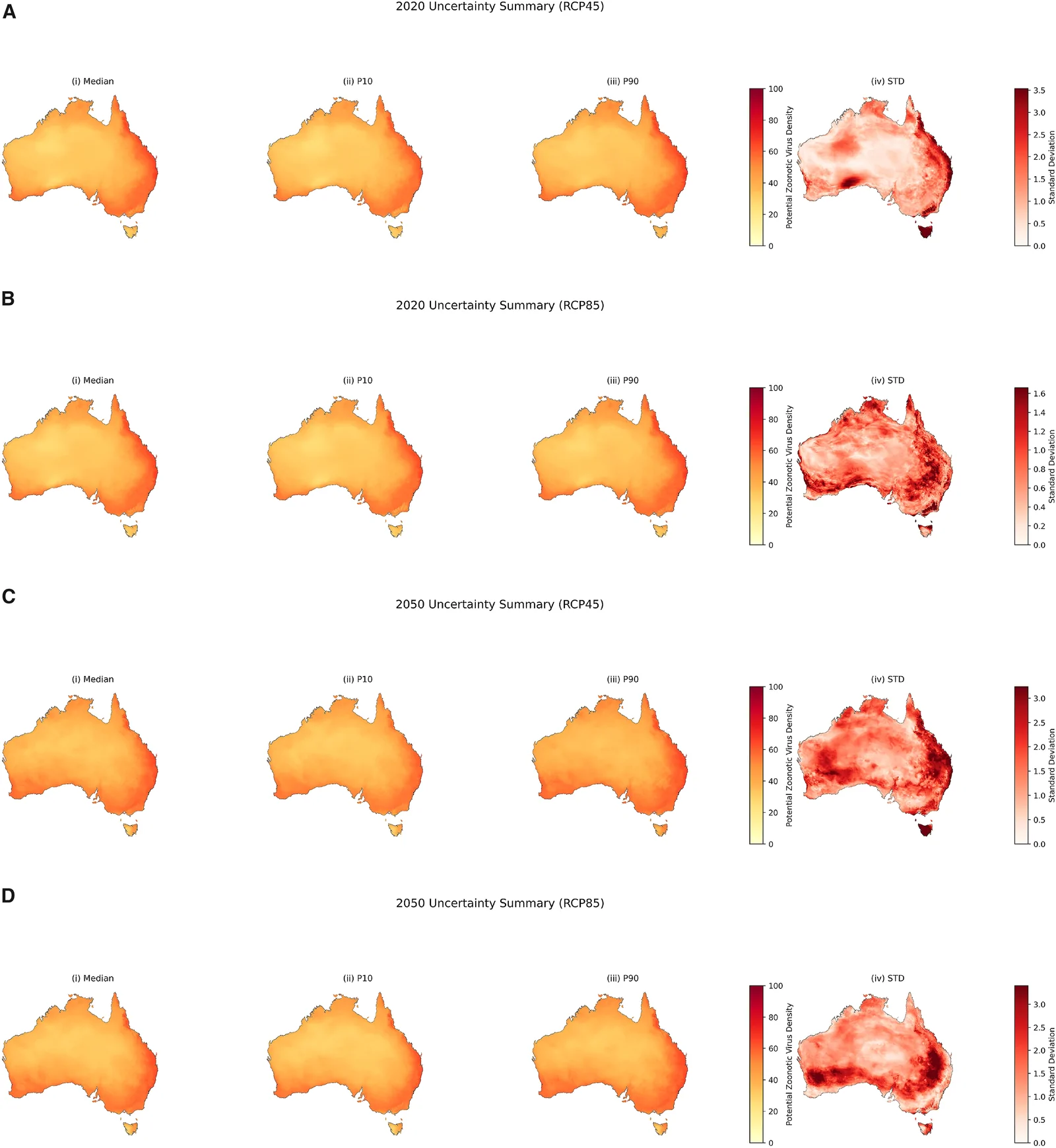
Climate-model uncertainty in projected climate suitability for potential zoonotic virus density Ensemble-based uncertainty summaries of projected climate suitability for potential zoonotic virus density across Australia for 2020 under (A) RCP 4.5 and (B) RCP 8.5 and 2050 under (C) RCP 4.5, and (D) RCP 8.5. For each scenario × year combination, images show (i) the ensemble median, (ii) 10^th^ percentile (*P*10), (iii) 90^th^ percentile (*P*90), and (iv) inter-model standard deviation (STD), calculated across all global climate models. Image (iv) shows where model disagreement is high.

Uncertainty summaries (Figure 6) showed that median, 10^th^-percentile, *P*10, and 90^th^-percentile, *P*90, projections had broadly similar spatial patterns across both RCP scenarios and time periods. In all cases, higher predicted potential zoonotic virus density was mainly concentrated along coastal regions, while lower values were more common across inland Australia. The standard deviation maps (image (iv)) showed that uncertainty was not evenly distributed across Australia. Higher uncertainty occurred mainly along parts of eastern Australia, southern coastal regions, southwestern Australia, and Tasmania. In contrast, much of the inland and central regions showed lower standard deviation values, indicating stronger agreement among the GCMs in these areas.

### Spatial clustering patterns are robust across climate models

**Figure 7:**
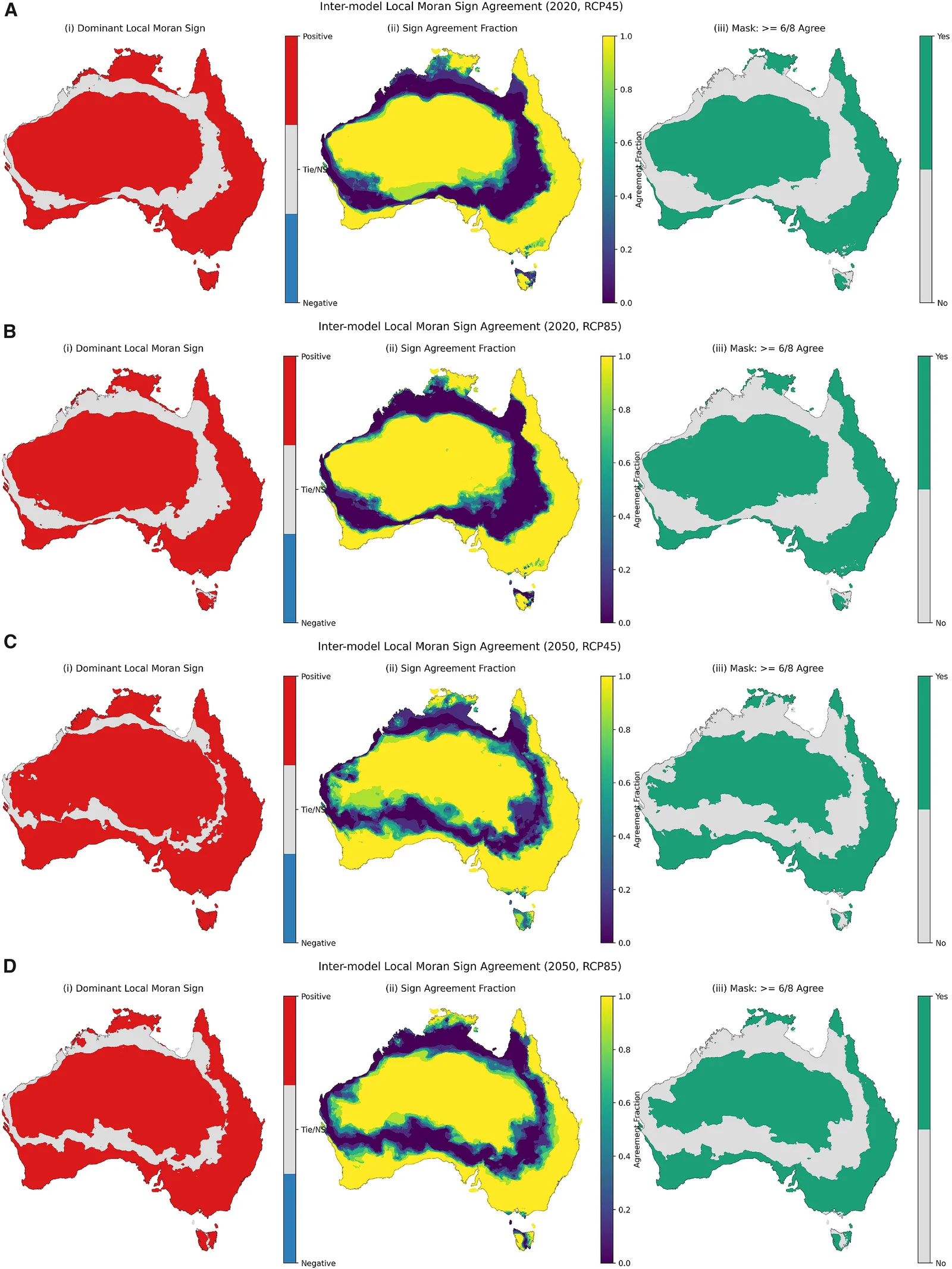
Spatial autocorrelation of projected climate suitability for potential zoonotic virus density Local indicators of spatial association (LISA) sign agreement for 2020 under (A) RCP 4.5 and (B) RCP 8.5, and for 2050 under (C) RCP 4.5, and (D) RCP 8.5. Image (i) shows the dominant local Moran sign across climate models (positive, negative, or tie/nonsignificant), image (ii) shows the fraction of models agreeing on the local Moran sign at each grid cell, and image (iii) shows a binary mask identifying locations where at least 6 of 8 climate models agree on the direction of spatial association. Detailed LISA cluster maps for each individual climate model are provided in Figures S5 and S6.

Moran’s I statistics reported here are used as a descriptive pattern diagnostic rather than an independent ecological validation of the projections. Spatial autocorrelation was evident across all projections, with global Moran’s I values consistently positive and statistically significant (mean ∼0.99). Local Moran’s I agreement maps showed that clustering patterns were broadly consistent across GCMs in both 2020 and 2050 projections and under RCP 4.5 and RCP 8.5 scenarios (Figure 7). High-density clusters were mainly located in coastal regions, including eastern, southeastern, northern, and southwestern Australia, whereas low-density clusters were concentrated across dry inland Australia. Inter-model agreement was generally high, with many regions showing agreement among six or more of the eight GCMs. Lower agreement occurred mainly in transition areas between coastal/high-density areas and inland/low-density areas where the local Moran’s I cluster category is nonsignificant. Local Moran’s I maps for all individual GCMs and scenarios are visualized in Figures S5 and S6.

### Relative humidity is the strongest climatic predictor

**Figure 8:**
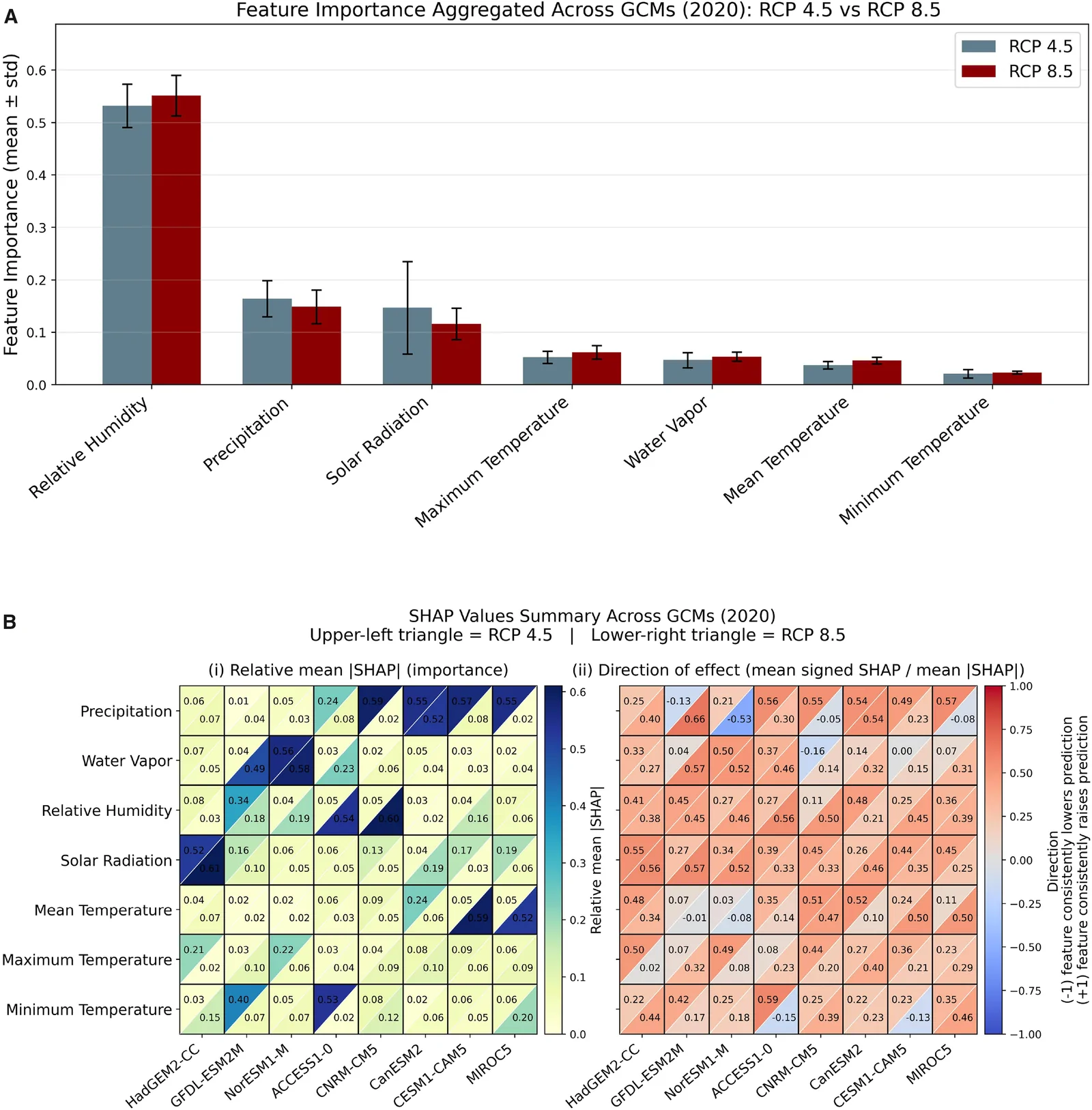
Aggregated climatic drivers of projected climate suitability for potential zoonotic virus density (A) Extra Trees Regressor feature importance values (mean ± standard deviation) aggregated across all eight global climate models (GCMs) for RCP 4.5 (dark gray) and RCP 8.5 (dark red). (B) SHAP summary across all eight GCMs showing the (i) mean absolute contribution, and (ii) direction of effect of each climate variable to model predictions under RCP 4.5 (upper-left triangle) and RCP 8.5 (lower-right triangle). See ETR feature importance by individual GCMs in Figure S7 and SHAP beeswarm feature importance for individual GCMs in Figure S8.

Feature importance analysis identified relative humidity as the strongest predictor of potential zoonotic virus density across the GCMs (Figure 8A), with mean importance values exceeding 0.5 for both emission scenarios. Precipitation and solar radiation showed moderate importance (mean importance value ∼0.1–0.2), whereas temperature-related variables contributed less overall (mean importance value < 0.1).

The SHAP summary plots (Figure 8B) showed that the relative influence of climatic predictors varied across GCMs and between RCP scenarios. In this figure, image (i) shows the relative mean absolute SHAP values, which represent climate variable importance. Higher values indicate that a variable had a stronger influence on model predictions. The upper left triangle of each cell represents RCP 4.5, while the lower right triangle represents RCP 8.5.

Across models, the dominant predictors differed. Solar radiation had the strongest SHAP importance in HadGEM2-CC, while water vapor was most important in NorESM1-M. Precipitation was the dominant predictor in CNRM-CM5, CanESM2, CESM1-CAM5, and MIROC5, particularly under the RCP 4.5 scenario. Relative humidity showed low to moderate SHAP importance across several models and was less dominant than its ETR feature importance in Figure 8A. This indicates that although relative humidity produced substantial impurity reduction across the fitted trees, other climatic variables made stronger local contributions to prediction values in some GCMs.

Image (ii) shows the direction of each predictor’s effect, calculated as the mean signed SHAP value divided by the mean absolute SHAP value. Positive values indicate that a variable generally increased predicted potential zoonotic virus density, while negative values indicate that it generally reduced predictions. Most predictors had positive direction values across the GCMs, suggesting that on average, higher values of these climate variables generally increased predicted zoonotic virus density. However, some negative or near-zero values were present, especially for precipitation and temperature-related variables in selected models, showing that predictor effects were not fully consistent across all GCMs.

## Discussion

This study demonstrates that climate-driven changes in potential zoonotic virus density across Australia are spatially structured, robust across climate models, and strongly shaped by hydrological variables, particularly relative humidity. By integrating an ensemble of eight GCMs with spatially explicit projections, we show that future climatic change is likely to redistribute ecological conditions associated with viral diversity rather than produce uniform increases in hazard across Australia.

### Model performance and spatial generalization

Overall model performance was moderate to strong across most GCM × RCP combinations, with test R^2^ values ranging between ∼0.69 and 0.73 and relatively consistent RMSE and MAE values across models. This suggests that the ETR captured a substantial proportion of the spatial variation in potential zoonotic virus density. However, performance was not identical across models (Figure S2). GFDL-ESM2M under RCP 4.5 was a clear lower-performing outlier, with reduced R^2^ (0.6) and higher RMSE and MAE compared with the other model-scenario combinations. This indicates that although ensemble-level patterns are robust, some individual GCM outputs should be interpreted with greater caution.

### Spatial structure of projected virus density

Across all GCMs and emission pathways, projected climatic suitability for potential zoonotic virus density shows strong and persistent spatial autocorrelation. High-density clusters are consistently identified across eastern, southeastern, and northern coastal Australia, as well as parts of southern and southwestern Australia, while lower density values are concentrated throughout dry and semi-dry inland Australia. This spatial structure is also reflected in the local Moran classifications, where high-high clusters occur mainly around coastal areas, and low-low clusters dominate much of inland Australia (Figures S5 and S6).

The local Moran agreement summaries (Figure 7) show that the sign of spatial autocorrelation is broadly consistent across models and RCPs. Large areas of Australia show agreement among at least six of the eight GCMs, particularly in the interior low-density areas and the coastal high-density areas. Disagreement is concentrated mainly in transitional regions between these two areas, especially where small changes in climate can shift a grid cell between cluster classes. This suggests that the highest uncertainty is where spatial regimes change.

### Redistribution and homogenization rather than uniform increase

A key finding is that projected change from 2020 to 2050 is spatially heterogeneous (Figures 2C and 3C). The change maps do not show a uniform increase across Australia. Instead, increases are concentrated in parts of western, southwestern, southern inland, and some central areas, while decreases are more evident across portions of eastern and northeastern Australia. This pattern is important because it suggests that climate change may redistribute ecological suitability for zoonotic virus density rather than amplifying current hotspots.

The ensemble median change maps (Figures 4A and 4B, image (i)) reinforce this interpretation. Under both RCP 4.5 and RCP 8.5, positive changes are most visible across parts of inland and southwestern Australia, whereas negative changes occur across areas that currently exhibit high density, particularly in eastern Australia. This produces a partial smoothing of the current spatial projections: some lower-density regions increase, while some higher density regions decrease.

## Differences between RCP 4.5 and RCP 8.5

The broad spatial patterns between RCP 4.5 and RCP 8.5 are similar; however, RCP 8.5 showed slightly stronger local contrasts in the 2050–2020 change maps and in some areas of inter-model disagreement, particularly across transitional areas between inland low-density regions and coastal or southeastern higher density areas (Figures 2C, 3C, 4A, 4B, 6C, and 6D).

The density bin results (Figure 5 and Table S2) show that in 2020, most pixels fall within the 20–40 and 40–60 density classes, with only a small proportion in the 60–80 class and essentially none in the extreme 0–20 or 80–100 bins. By 2050, the distribution shifts further toward intermediate values (40–60). Under RCP 4.5, the average proportion of pixels in the 40–60 class increases to approximately half of the landscape (∼49.3%), while the 20–40 class declines by approximately 12.5%. Under RCP 8.5, the 20–40 class remains slightly more dominant than under RCP 4.5, but the 40–60 class still increases by approximately 8.3% relative to 2020. The 60–80 class remains small under both scenarios and does not expand substantially.

This is an important result because it means that higher emissions do not simply translate into more pixels in the highest density classes. Instead, both RCPs suggest a movement toward midrange suitability, with relatively few areas reaching very high predicted density. This supports the interpretation that climate change is reshaping the spatial distribution of suitability rather than causing a uniform escalation of potential zoonotic virus density.

### Inter-model agreement and disagreement

The inter-model agreement maps (Figures 4A and 4B, images (ii)–(iv)) show that the direction of projected change is relatively consistent across much of Australia. Strong agreement occurs across large parts of western, central, and northern Australia, whereas disagreement is concentrated mainly in transitional regions along eastern and southeastern Australia, with smaller localized areas in the north. These disagreement areas identify regions where projected results are sensitive to GCM projections. These are the areas where additional climate model uncertainty should be considered before drawing strong conclusions about future zoonotic hazard.

The disagreement maps also show that the largest absolute changes at disagreement pixels are generally low compared with the full range of predicted density. This suggests that disagreement is often concentrated around marginal shifts rather than extreme changes. Therefore, while the precise direction of change may be uncertain in some transition areas, the broader conclusion of spatial redistribution remains robust.

### Uncertainty patterns

The uncertainty summaries (Figure 6, image (iv)) show spatial variability in uncertainty among the eight GCM projections. Areas with low standard deviation indicate locations where the climate models predict similar estimates of potential zoonotic virus density, suggesting higher confidence in these patterns, whereas areas with high standard deviation indicate stronger inter-model disagreement. This highlights regions where projections are more sensitive to differences in climate model structure, particularly in hydrological variables. Higher uncertainty occurred along parts of eastern Australia, northern coastal areas, Tasmania, and some southwestern and southern regions. In contrast, parts of the dry interior show relatively low uncertainty, consistent with low estimates of projected density. This suggests that uncertainty is highest where climate-driven suitability is most spatially variable or where different GCMs produce different local climate responses.

### Climatic drivers of projected density

Feature importance analyses identify relative humidity as the dominant predictor in the ETR models across both RCPs (Figure 8A). This pattern is highly consistent in the individual model feature importance plots, where relative humidity usually explains the largest share of model behavior (Figure S7). Precipitation and solar radiation generally act as secondary predictors, while temperature variables contribute less overall.

However, the SHAP results (Figures 8B and S8) add an important layer of interpretation. While ETR feature importance highlights relative humidity as the dominant global predictor, SHAP values reveal stronger model-specific and context-dependent effects. In some GCMs, precipitation, solar radiation, or water vapor show large local contributions, even when relative humidity remains dominant in the global importance ranking. This difference is because tree-based feature importance summarizes how often and how effectively a variable is used for splitting across the model, whereas SHAP values describe how much each variable contributes to individual predictions. The two approaches together indicate that moisture availability is the main climatic variable structuring potential zoonotic virus density. The SHAP beeswarm plots also suggest that the influence of a variable changes depending on its value and regional climatic context.

### Interpretation of increases in dry and inland regions

The projected increases in dry and semi-dry western, central, and southwestern Australia suggest that future climate conditions in some inland areas may become more suitable for zoonotic viruses than they are today. This is likely because changes in moisture-related variables, such as humidity or rainfall, may make these dry regions more similar to areas where higher virus density is currently found. However, this does not mean that many new host species will suddenly move into these regions, or that spillover risk will immediately increase. Therefore, the predicted increases should be interpreted as changes in climatic suitability, not as direct evidence of new host assemblages or immediate disease risk. These inland areas may still be important because they are often less studied than coastal hotspots. Future field sampling of hosts and viruses in these areas would help test whether the model predictions are realistic.

### Spatial autocorrelation and ecological clustering

The local Moran analyses (Figures S5 and S6) provide additional evidence that projected virus density is spatially clustered. High-high and low-low clusters dominate the classification maps, while high-low and low-high outlier classes are negligible. This indicates that potential zoonotic virus density is not distributed as isolated pixels, but as broad spatial regimes. The transition from 2020 to 2050 modifies the boundaries of these regimes. Low-low clusters remain extensive across the inland, but their edges shift in some models. High-high clusters remain associated with coastal regions, although some high-density areas show reductions in projected density by 2050. The gray nonsignificant areas are also informative because they mark areas where spatial clustering is weaker or more transitional. These areas are more likely to be sensitive to climate model uncertainty.

### Implications for surveillance and public health interpretation

It is critical to emphasize that potential zoonotic virus density represents an early ecological hazard indicator rather than a direct measure of spillover risk or human disease incidence. Potential zoonotic virus density reflects climatic suitability associated with known host and virus relationships and host distributions, but it does not include human exposure, land use, livestock density, pathogen transmissibility, healthcare access, or behavioral factors. An area with higher potential zoonotic virus density is not necessarily an area with higher immediate human disease risk.

Translating these projections toward operational risk intelligence would require several additional steps. Overlaying potential zoonotic virus density projections with human population density and wildlife-human interface data would identify regions where ecological hazard and human exposure co-occur. Incorporating dynamic host distribution models would capture climate-driven range shifts not represented in the current fixed baseline. Finally, weighting the potential zoonotic virus density surface by pathogen-specific characteristics (such as host range breadth and documented human infectivity) would help prioritize surveillance toward the highest-consequence ecological zones. These extensions would move the current ecological hazard baseline toward an integrated One Health risk assessment framework.

Nevertheless, the projections are useful for identifying where ecological conditions associated with zoonotic viral diversity may shift under climate change. The results suggest that surveillance strategies should not focus only on current high-density coastal regions. They should also consider inland and southwestern areas where climatic suitability may increase, as well as transition areas where model uncertainty is high. These areas may be particularly valuable for early ecological monitoring because they represent areas where climate-driven changes could alter host and virus dynamics before clear public health signals emerge.

### Limitations of the study

Several limitations should be considered when interpreting these findings. First, we assume a fixed host-virus-climate relationship and only implicitly capture shifts in host distributions. The baseline potential zoonotic virus density surface is based on contemporary host range information, but host species may shift their distributions in response to climate change, land use change, habitat modification, and biotic interactions. Incorporating dynamic species distribution models for major reservoir groups would improve the biological realism of future projections.

Second, known host and virus associations are incomplete and spatially biased. Viral discovery is much more intensive in some regions, host groups, and pathogen families than in others. This may lead to underestimation of potential zoonotic virus density in poorly sampled regions. Although the consistency of broad spatial patterns across climate models suggests that the main conclusions are robust, the absolute predicted values should be interpreted cautiously.

Third, uncertainty in the baseline virus density layer was not fully propagated through the modeling framework. The uncertainty maps capture variation across GCM projections, but they do not include uncertainty associated with host range maps, viral association data, detection bias, or model structure. Future work should aim to propagate these uncertainties through the full workflow.

Fourth, the analysis focuses on climate variables and does not include land use change, host abundance, species interactions, livestock systems, human population density, or mobility. These factors are essential for translating ecological hazard into spillover risk. Future extensions should integrate climatic suitability with ecological, demographic, and land use predictors to produce a more complete assessment of zoonotic emergence risk.

A further limitation is that the ETR models were trained on 2020 climate conditions and projected onto 2050 climate values. Where future climate inputs fall outside the range of the training data, model predictions represent extrapolations beyond observed conditions. Although tree-based methods constrain predictions within the range of training outputs, individual predictor values, particularly for moisture-related variables under RCP 8.5, may exceed training bounds in some regions. The degree of extrapolation was not formally quantified in this study and represents an additional source of uncertainty that should be assessed in future work, for example using multivariate environmental similarity surfaces (MESS) or equivalent approaches.

Finally, the model was trained exclusively on a single projected 2020 potential zoonotic virus density surface and has not been temporally validated against historical host distribution or virus detection records. Therefore, the 2050 outputs are mathematically re-projected spatial patterns of climate suitability under future climate scenarios.

## Resource availability

### Lead contact

Requests for further information and resources should be directed to and will be fulfilled by the lead contact, Prof. Roslyn I. Hickson (roslyn.hickson@csiro.au).

### Materials availability

This study did not generate new unique reagents.

### Data and code availability

• Raw CMIP5 climate projections are publicly available from the NCI Australia Climate Change in Australia portal (http://www.climatechangeinaustralia.gov.au/).
• Models’ inputs and outputs GeoTIFF rasters for all GCM × RCP × year combinations are stored within the CSIRO data hub and are available at https://doi.org/10.25919/kb5g-5b89.
• All original codes have been deposited at Zenodo at https://doi.org/10.5281/zenodo.20502428.
• Any additional information required to reanalyze the data reported in this paper is available from the lead contact upon request.

## Acknowledgments

We acknowledge the World Climate Research Programme’s Working Group on Coupled Modelling, which is responsible for CMIP, and we thank the climate modeling groups (listed in the Introduction section of this paper) for producing and making available their model output. For CMIP, the U.S. Department of Energy’s Program for Climate Model Diagnosis and Intercomparison provides coordinating support and led development of software infrastructure in partnership with the Global Organization for Earth System Science Portals.

## Author contributions

Conceptualization, M.G., R.I.H., and J.D.S.; data curation, M.G. and J.D.S.; formal analysis, M.G. and R.I.H.; investigation, M.G., R.I.H., and J.D.S.; methodology, M.G., R.I.H., and J.D.S.; software, M.G. and J.D.S.; validation, M.G., R.I.H., and J.D.S.; visualization, M.G., R.I.H., and J.D.S.; writing – original draft, M.G., R.I.H., and J.D.S.; writing – review and editing, M.G., R.I.H., and J.D.S.

## Declaration of interests

The authors declare no competing interests.

## Declaration of generative AI and AI-assisted technologies in the writing process

During the preparation of this work, M.G. and R.I.H. used Claude (Sonnet 4.6) and M365 Copilot for the purposes of language editing (clarity, grammar, and readability) of text written by the author, and not for scientific analysis, reasoning, or interpretation. After using this tools or services, the authors reviewed and edited the content as needed and take full responsibility for the content of the publication.

## STAR★Methods

### Key resources table

**Table:** 

REAGENT or RESOURCE	SOURCE	IDENTIFIER
**Deposited data**		

Potential zoonotic virus density (2020 baseline)	Golchin et al.^9^	https://doi.org/10.1016/j.onehlt.2024.100737
Gridded climate projections (CMIP5; RCP 4.5 and 8.5)	Australian Government – Climate Change in Australia (CCiA) portal^21^	https://www.climatechangeinaustralia.gov.au
IUCN Red List species range maps (version 2022)	IUCN (2022)^22^	https://www.iucnredlist.org
Analysis code	This paper	https://doi.org/10.5281/zenodo.20502428
Model inputs and outputs	This paper	https://doi.org/10.25919/kb5g-5b89

**Software and algorithms**		

Python 3.11.6	Python Software Foundation	https://www.python.org/
scikit-learn v1.3.1	Pedregosa et al.^23^	https://scikit-learn.org
shap v0.45.1	Lundberg and Lee^24^	https://github.com/shap/shap
LazyPredict v0.2.12	Rao Pandala^25^	https://github.com/shankarpandala/lazypredict
PySAL v24.7	Rey and Anselin^26^	https://pysal.org
esda v2.6.0	PySAL Developers	https://github.com/pysal/esda

### Method details

All analyses were performed in Python (version 3.11.6). Machine learning models were implemented using “scikit-learn” (version 1.3.1), with model interpretation conducted using “shap” (version 0.45.1). Spatial analyses, including Moran’s I and Local Indicators of Spatial Association, were performed using “PySAL” (version 24.7) and “esda” (version 2.6.0).

#### Climate data

Gridded climate projections were obtained from the Australian Government’s Climate Change in Australia (CCiA) portal.^21^ Climate variables were extracted for the baseline year (2020) and a future projection year (2050) under two Representative Concentration Pathways (RCP 4.5 and RCP 8.5) and eight global climate models (GCMs): MIROC5, HadGEM2-CC, GFDL-ESM2M, CanESM2, CNRM-CM5, CESM1-CAM5, NorESM1-M, and ACCESS1-0. All climate data were supplied in NetCDF format, referenced to the WGS84 geographic coordinate system (EPSG:4326), at a spatial resolution of 5 km and monthly temporal resolution. Climate variables included minimum, mean, and maximum air temperature, relative humidity, precipitation, solar radiation, and water vapor. These variables were selected based on their availability across all GCM-RCP combinations and used as the feature sets to train the machine learning models.

#### Target/baseline variable: potential zoonotic virus density

The response variable used for model training was the spatial estimate of potential zoonotic virus density developed by Golchin et al.^9^ for the year 2020 (Figure S1). This variable represents a proxy indicator of spillover hazard by estimating the spatial density of animal viruses with zoonotic potential, rather than the realized zoonotic transmission or outbreak occurrence. It captures the spatial concentration of wildlife-associated viruses with zoonotic potential. As such, this variable reflects hazard presence rather than a prediction of spillover events or disease incidence.^4^^,^^8^^,^^27^ In this study, this spatial surface was held fixed over the projection period and used as the baseline ecological target for climate-driven modeling.

Golchin et al.^9^ derived potential zoonotic virus density by integrating:

(1) International Union for Conservation of Nature (IUCN) Red List species range maps (version 2022)^22^ for eight mammalian and three avian orders (*Rodentia*, *Primates*, *Perissodactyla*, *Lagomorpha*, *Diprotodontia*, *Chiroptera*, *Cetartiodactyla*, *Carnivora*, *Passeriformes*, *Galliformes*, and *Anseriformes*);
(2) host and virus association data and order-specific proportions of viruses with zoonotic potential from Mollentze and Streicker.^28^

For each grid cell, the expected contribution of zoonotic viruses from all overlapping host species was summed to produce a gridded viral hazard surface at 0.01 ° × 0.01 ° spatial resolution (approximately 1 km × 1 km).

#### Data processing and harmonization

Monthly climate data for each variable were aggregated to a single annual value per grid cell using the median across months. The median aggregation was selected to reduce sensitivity to extremes and outlier months, better represent typical annual conditions, and to be consistent with the nature of the baseline variable. All climate data were converted to GeoTIFF format and resampled to 0.05 ° × 0.05 ° (approximately 5 km × 5 km) using bilinear interpolation. The potential zoonotic virus density raster was subsequently re-projected, resampled, and spatially aggregated by summation to preserve total viral density within each coarser grid cell and ensure alignment with climate predictors. Climate predictors and the response variable were spatially joined. Grid cells containing missing values in any predictor variable were removed from the dataset.

To reduce spatial leakage between training and testing data, samples were divided using a checkerboard spatial blocking approach. Latitude and longitude coordinates were first assigned to fixed geographic grid cells based on the 5° grid size (approximately 500 km), and each grid cell was given a unique spatial block identifier. Blocks were then divided into two alternating sets to build the training and testing sets, producing an alternating checkerboard pattern across Australia.

All predictor variables were standardized using a *Z* score transformation (mean = 0, standard deviation = 1) implemented using the “StandardScaler” class in “scikit-learn”.

#### Model selection

An initial comparison of candidate regression algorithms was conducted using the “LazyRegressor” (“LazyPredict” v0.2.12).^25^ Candidate models included Extra Trees Regressor (ETR), Random Forest, Bagging, Decision Tree, Gradient Boosting, and AdaBoost Regressors, all implemented using the “scikit-learn” package.

Models were evaluated using a random 70/30 train-test split based on adjusted R^2^, R^2^, root-mean-square error (RMSE), and computational runtime, using climate data from the ACCESS1-0 GCM under RCP 4.5 as a representative training set. ACCESS1-0 was selected at this stage because it is the Australian-developed CMIP5 global climate model and this study focuses on climate-driven projections across Australia. Model selection was exploratory and intended to identify an appropriate algorithm rather than to compare climate model performance. Based on this preliminary comparison, the ETR showed the best performance and was selected for all subsequent analyses.

#### Extra trees model specification and training

The ETR^29^ is an ensemble tree-based method that builds multiple decision trees using randomized feature splits and averages their predictions across the ensemble. This added randomness helps reduce overfitting compared with a single decision tree, making the method well suited to spatially structured environmental data.

Hyperparameters were optimized using randomized search with 50 trials and 5-fold spatially informed cross-validation. The search explored variation in the number of trees ([100, 150, 200]), maximum tree depth ([2, 5, 10]), minimum samples required to split an internal node ([2, 4, 6]), minimum samples required at each leaf node ([1, 2, 3, 4]), and feature subsampling strategy ([None (i.e., all predictor variables), “sqrt”, “log2”]). Separate ETR models were tuned independently for each GCM × RCP combination using 2020 climate predictors and the baseline potential zoonotic virus density surface, resulting in 16 fitted models. These models were then used to generate spatial projections of potential zoonotic virus density for 2020 and 2050. Optimized ETR hyperparameters are provided in Table S1.

#### Projection comparison and climate model uncertainty

Model projections of potential zoonotic virus density were generated for 2020 and 2050 for each GCM and RCP combination using the trained ETR models. For each model and scenario combination, temporal change was quantified by subtracting the 2020 prediction from the corresponding 2050 projection, producing spatial difference maps of projected climate-driven change.

To summarize uncertainty across climate models, predictions were stacked across the eight GCMs for each RCP and year. Ensemble statistics were then calculated at each pixel, including the median, 10^th^ and 90^th^ percentiles, and standard deviation as central tendency, uncertainty bounds, and inter-model variability, respectively.

To assess spatial redistribution in projected density values, predictions were also categorized into five fixed density bins: [0–20], (20–40], (40–60], (60–80], and (80–100]. For each year, RCP and GCM, the number and percentage of valid pixels within each bin were calculated to compare shifts in the distribution of projected climate suitability for potential zoonotic virus density across models, scenarios, and time periods.

### Quantification and statistical analysis

#### Spatial autocorrelation analysis

Spatial clustering in projected climate suitability for potential zoonotic virus density was assessed using global and local Moran’s I statistics.^30^^,^^31^ These analyses were conducted for each GCM × RCP × year combination to answer two questions: whether projected density values were spatially clustered globally, and where localized clusters or spatial outliers occurred.

Spatial weights were defined using an eight-nearest-neighbor structure, approximating the eight cell Moore neighborhood of a regular grid. The weights matrix was standardized across rows to account for local differences in neighbor relationships. Global Moran’s I was then calculated for projected climate suitability of potential zoonotic virus density to quantify whether values showed overall spatial clustering, spatial randomness, or spatial dispersion. Local Moran’s I was calculated using 999 permutations to identify localized High-High (when a high value surrounded by neighbors with high values), Low-Low (when a low value surrounded by neighbors with low values), Low-High (when a low value surrounded by high value neighbors), and High-Low (when a high value surrounded by low value neighbors) spatial associations. Local clusters with pseudo-p-values greater than 0.05 were classified as nonsignificant.

#### Feature importance and SHAP analysis

To interpret the climatic drivers of projected climate suitability for potential zoonotic virus density, variable importance was assessed using two complementary approaches. First, feature importance scores were extracted from each fitted ETR model, based on impurity reduction across the ensemble. Second, SHapley Additive exPlanations (SHAP)^24^ were calculated to evaluate the direction and magnitude of each climate variable’s contribution to individual model predictions.

Feature importance and SHAP analyses were performed separately for each GCM × RCP combination for the 2020 fitted models. For each model, the fitted scaler from the modeling pipeline was used to transform the predictor variables before SHAP values were calculated with TreeExplainer. To reduce computational cost, grid cells were stratified into ten brackets based on predicted potential zoonotic virus density, and up to 100 grid cells were sampled from each bracket. SHAP values were then calculated for this stratified sample.

To assess consistency across climate models and emissions pathways, feature importance and SHAP outputs were aggregated across the eight GCMs. Aggregated summaries included mean ± standard deviation feature importance, mean absolute SHAP importance, and the average direction of effect.

## Supplemental information

Document S1. Figures S1–S8 and Tables S1 and S2 and supplemental references
