# Supplementary material for: Modeling climate-driven changes in potential zoonotic virus density across Australia
Source: iScience. 2026 Sep 1;29(9):117414. doi: 10.1016/j.isci.2026.117414 (PMC13571117; doi:10.1016/j.isci.2026.117414)
Supplement: Supplementary file 1 [file mmc1.pdf]

iScience, Volume *29*

Supplemental information

Modeling climate-driven changes in potential zoonotic virus density across Australia

Maryam Golchin, Justin D. Sexton, and Roslyn I. Hickson

Document S1. Supplemental Figures, Supplemental Tables, and Supplemental References

The analyses presented in this document extend the methods described in the main text and STAR Methods by providing per model outputs, diagnostic metrics, and uncertainty decompositions. All supplementary results are derived from the same trained Extra Trees Regressor models described in the main manuscript and correspond to the full ensemble of eight global climate models under RCP 4.5 and RCP 8.5.

SUPPLEMENTAL FIGURES

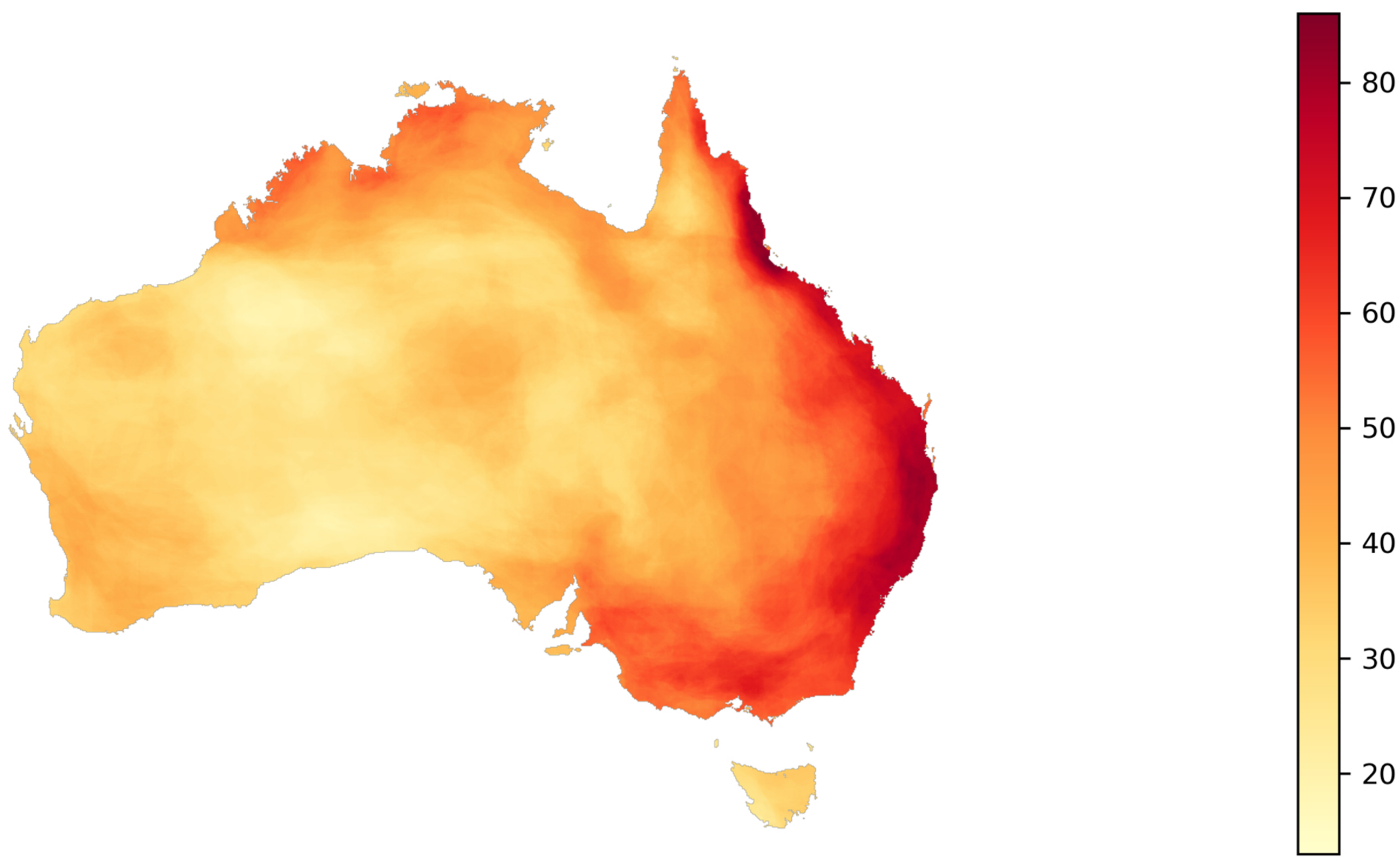

Figure S1. Baseline potential zoonotic virus density across Australia (2020), related to STAR Methods
Spatial distribution of estimated potential zoonotic virus density across Australia for the year 2020. Estimates were generated following the methodology of Golchin et al. [S1], integrating contemporary mammalian host species distributions and documented host and virus associations to quantify the ecological capacity of each grid cell to harbor zoonotic viruses. Warmer colors indicate higher estimated potential virus density. This baseline surface was used as the fixed target variable for all modeling analyses and as the reference state for climate-driven projections (see STAR Methods).

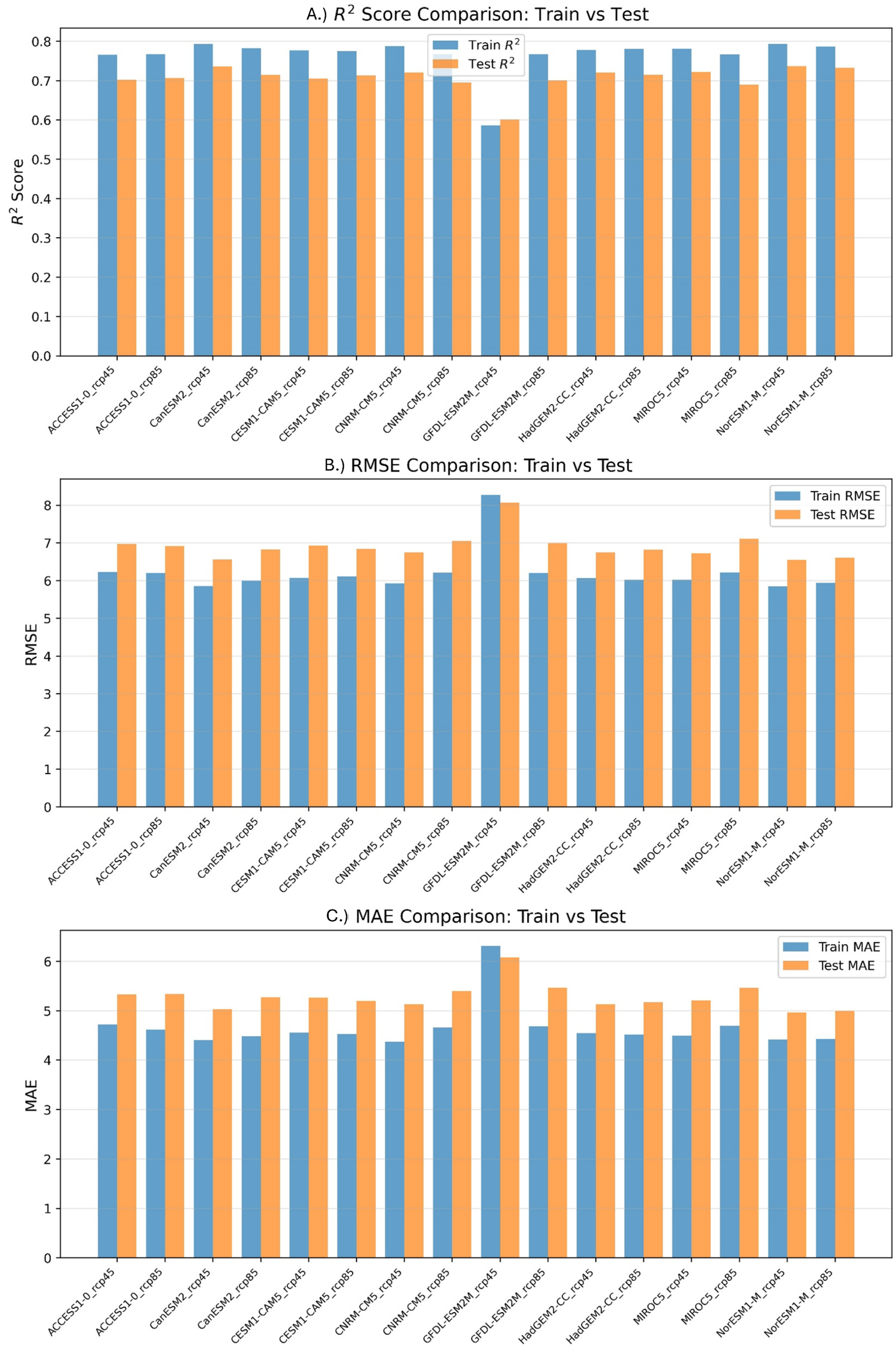

Figure S2. Model-level spatial cross-validation diagnostics, related to Figure 1

Comparison of training and testing performance metrics for the Extra Trees Regressor under checkerboard spatial block cross-validation across all global climate model (GCM) and representative concentration pathway (RCP) combinations.

A.) Coefficient of determination (R^2).

B.) Root mean square error (RMSE).

C.) Mean absolute error (MAE).

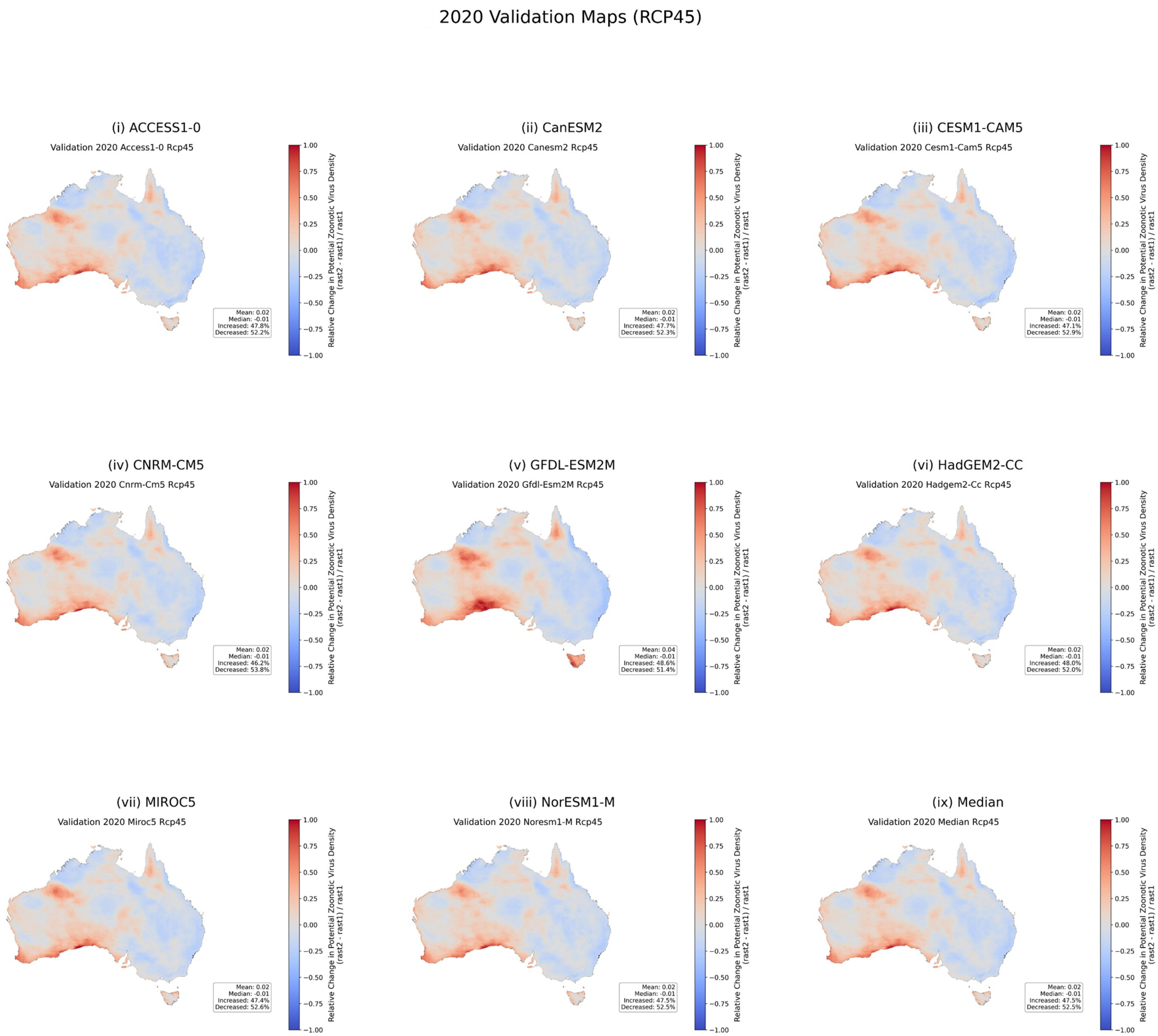

Figure S3. Spatial validation of cross-validated projections for 2020 under RCP 4.5, related to STAR Methods

Spatial distribution of relative cross-validated prediction residuals for potential zoonotic virus density in 2020 under RCP 4.5 emission scenario. Panels show validation maps for all eight global climate models as well as (ix) the ensemble median. The color scale ranges from -1 (blue, predicted value of zero) through 0 (grey, perfect prediction) to +1 (red, predicted double the target). Summary statistics shown in each panel report the mean and median residual values, along with the percentage of grid cells showing increases or decreases.

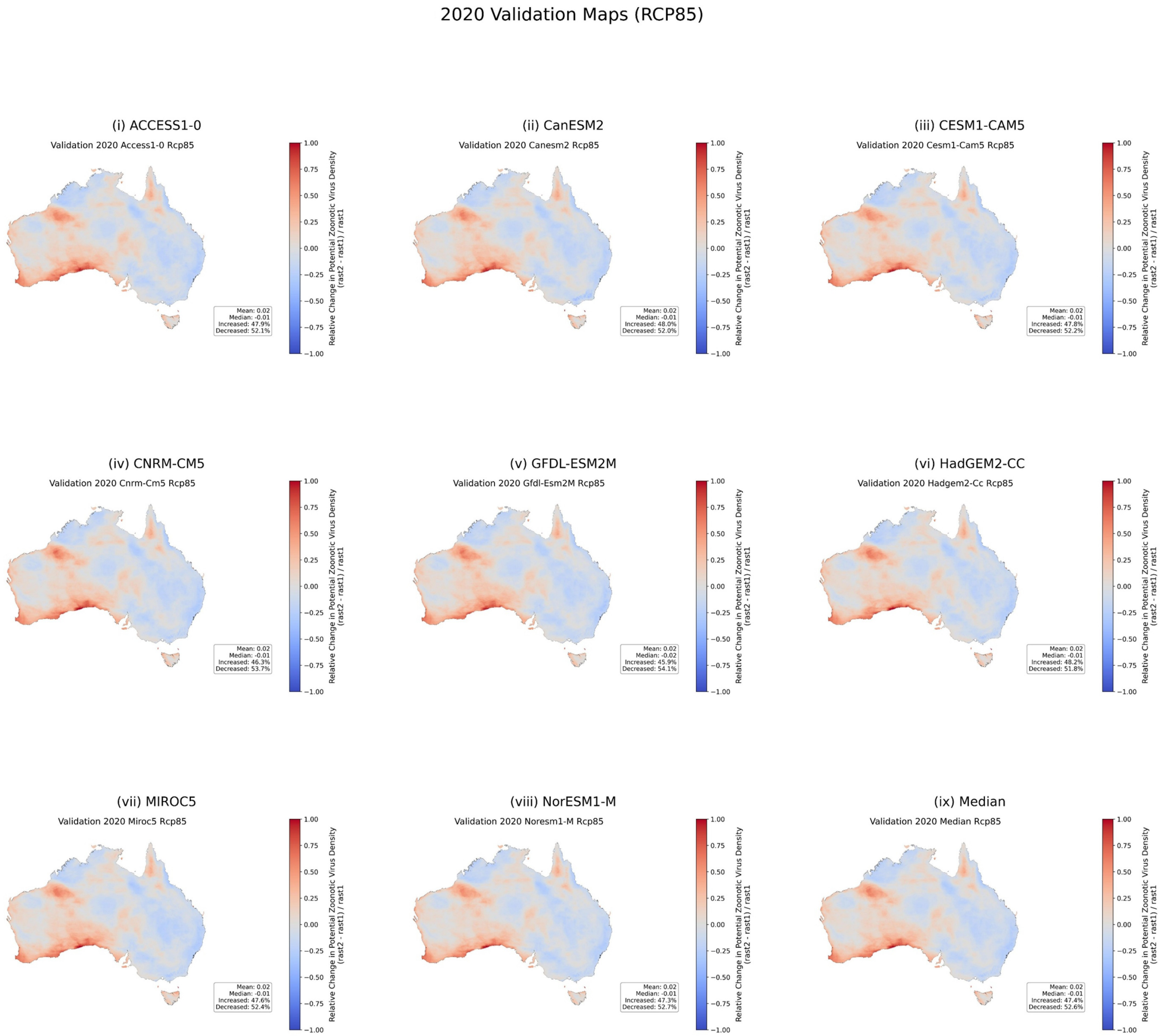

Figure S4. Spatial validation of cross-validated projections for 2020 under RCP 8.5, related to STAR Methods

Spatial distribution of relative cross-validated prediction residuals for potential zoonotic virus density in 2020 under RCP 8.5 emission scenario. Panels show validation maps for all eight global climate models as well as (ix) the ensemble median. The color scale ranges from -1 (blue, predicted value of zero) through 0 (grey, perfect prediction) to +1 (red, predicted double the target). Summary statistics shown in each panel report the mean and median residual values, along with the percentage of grid cells showing increases or decreases.

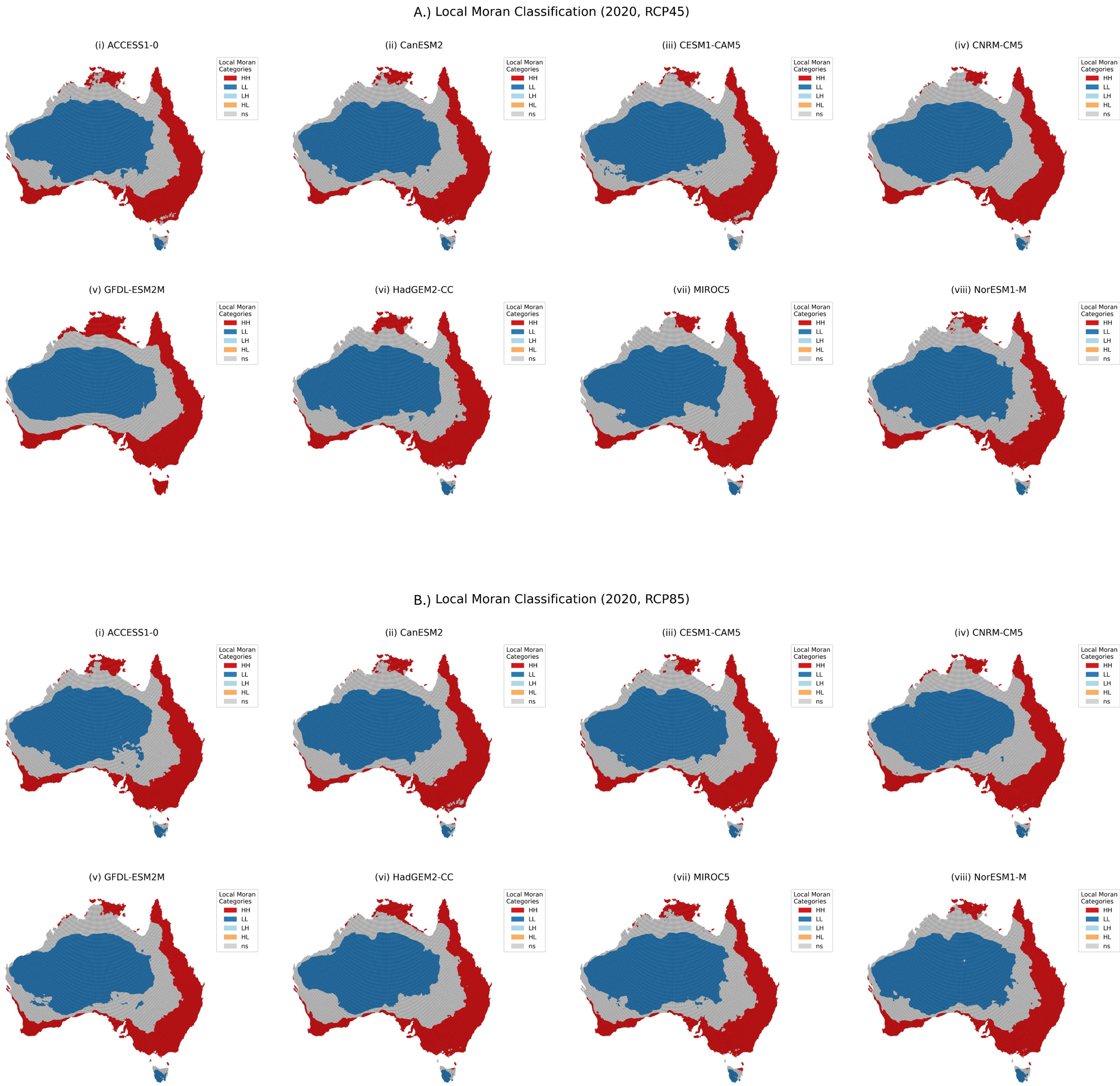

Figure S5. Local indicators of spatial association across climate scenarios for 2020, related to Figure 7

Local Indicators of Spatial Association cluster maps (p = 0.05) illustrating spatial autocorrelation patterns of projected potential zoonotic virus density across Australia. Panels show A.) RCP 4.5, and B.) RCP 8.5. Maps display localized High-High/HH (red), Low-Low/LL (dark blue), Low-High/LH (light blue), High-Low/HL (orange), and Not Significant/ns (gray) clustering patterns derived from Local Moran's I statistics calculated for each climate scenario and projection year. HL and LH spatial outlier classes were rare across all maps, with clustering dominated by HH and LL categories.

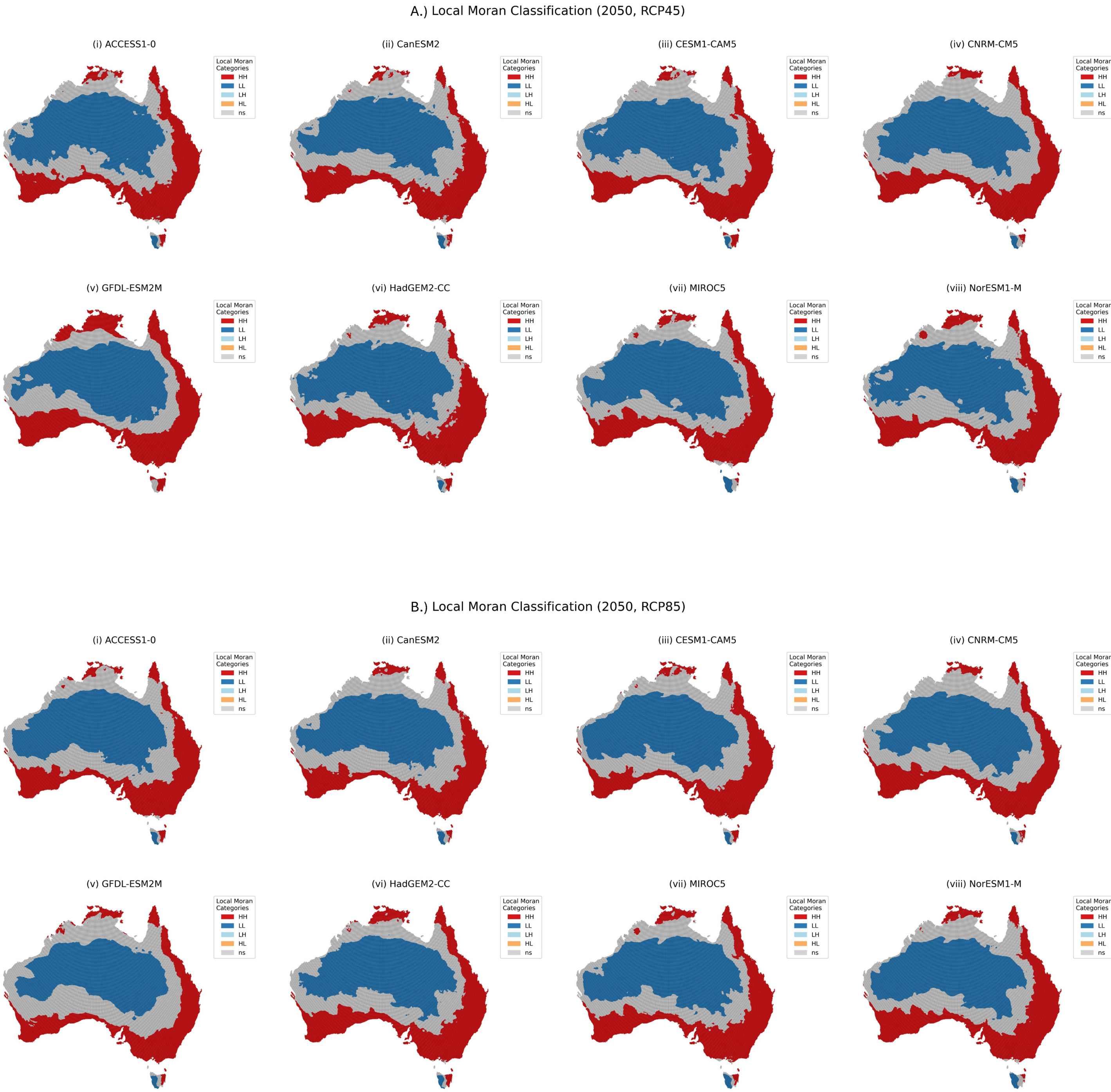

Figure S6. Local indicators of spatial association across climate scenarios for 2050, related to Figure 7

Local Indicators of Spatial Association cluster maps (p = 0.05) illustrating spatial autocorrelation patterns of projected potential zoonotic virus density across Australia. Panels show A.) RCP 4.5, and B.) RCP 8.5. Maps display localized High-High/HH (red), Low-Low/LL (dark blue), Low-High/LH (light blue), High-Low/HL (orange), and Not Significant/ns (gray) clustering patterns derived from Local Moran's I statistics calculated for each climate scenario and projection year. HL and LH spatial outlier classes were rare across all maps, with clustering dominated by HH and LL categories.

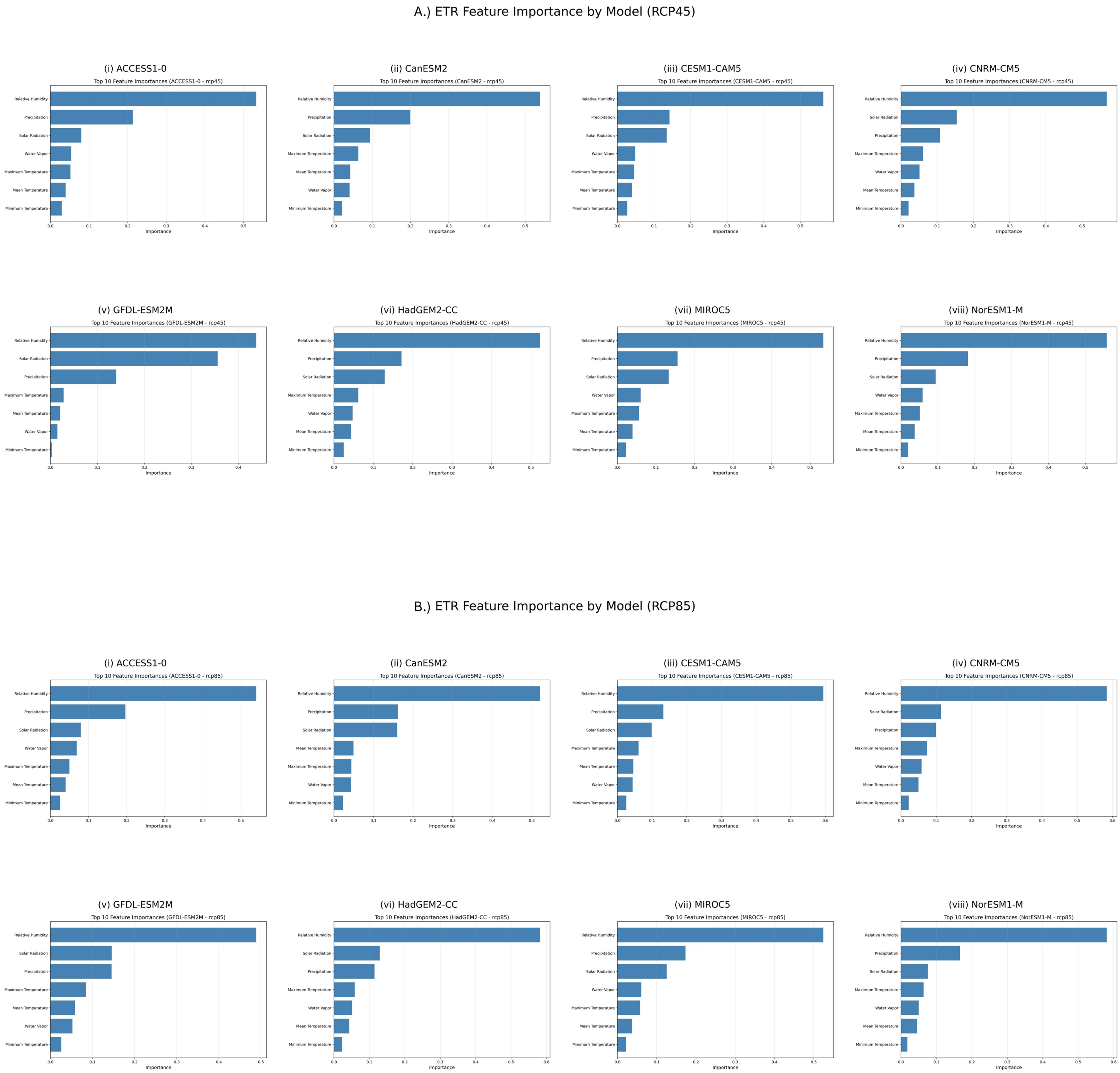

Figure S7. Model-specific Extra Trees Regressor feature importance, related to Figure 8a

Feature importance of climatic predictors in the Extra Trees Regressor for individual global climate models under A.) RCP 4.5 and B.) RCP 8.5. Feature importances were calculated using the fitted model for each GCM × RCP combination for the year 2020.

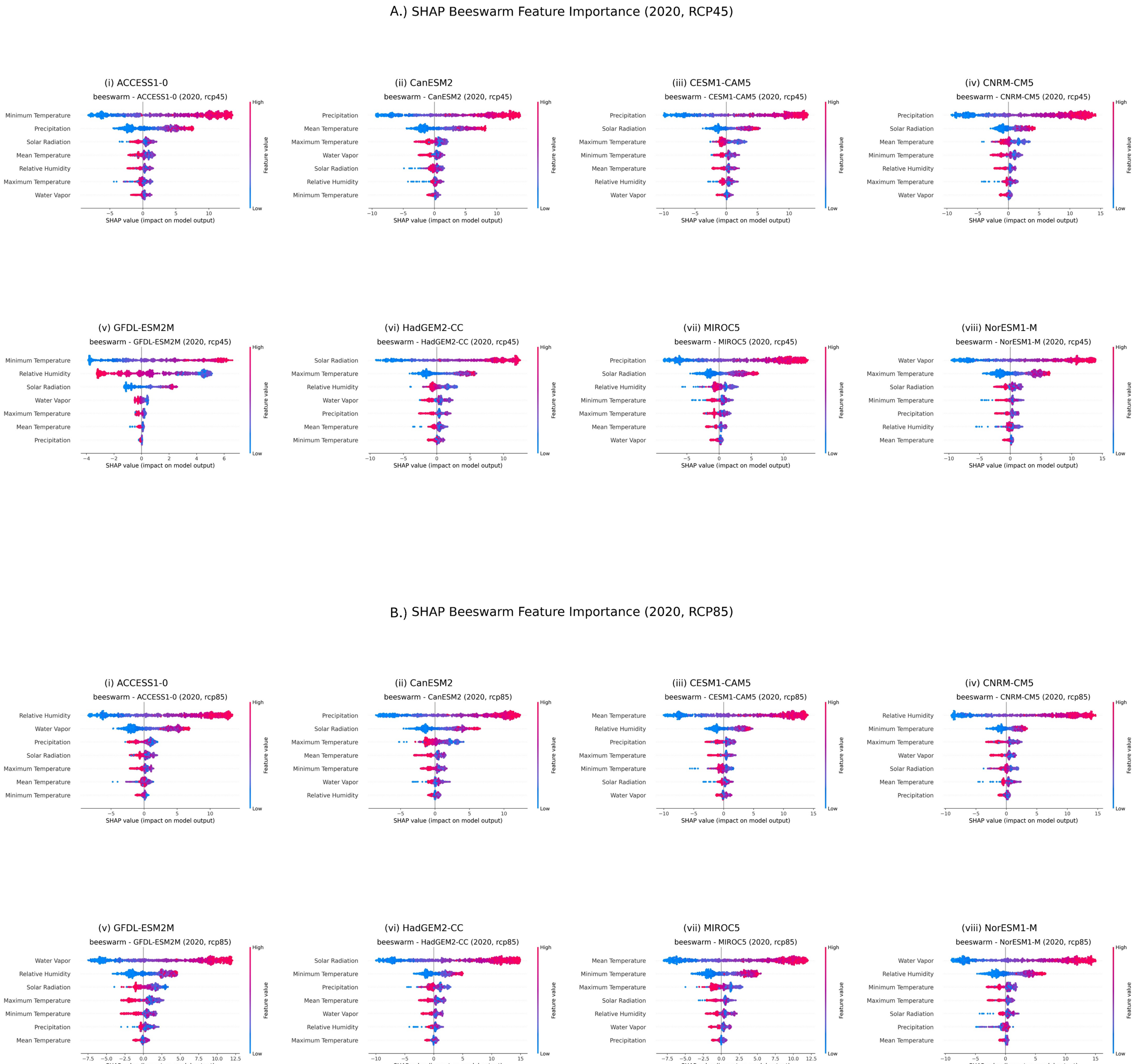

Figure S8. Model specific SHAP beeswarm plots illustrating climate variable contributions, related to Figure 8b

SHAP beeswarm plots showing the distribution of feature contributions to predicted potential zoonotic virus density for each global climate model under A.) RCP 4.5 and B.) RCP 8.5 for the year 2020. Each dot represents a single grid cell. The x-axis shows the SHAP value, indicating the direction and magnitude of a feature's contribution to predicted potential zoonotic virus density (positive values increase predicted potential zoonotic virus density; negative values decrease it). Features are ordered vertically by mean absolute SHAP value, with the most influential variables at the top. Dot color represents the original feature value, from low (blue) to high (red), allowing assessment of whether higher or lower values of a given climate variable are associated with positive or negative model contributions.

SUPPLEMENTAL TABLES

Table S1. Optimized Extra Trees Regressor hyperparameters, related to STAR Methods

Optimized hyperparameter values for the Extra Trees Regressor fitted separately for each global climate model and emission scenario. Reported parameters include number of estimators, maximum depth, minimum samples per split, minimum samples per leaf, and maximum features usage configuration.

Model	RCP	Number of estimators	Max depth	Minimum samples per split	Minimum samples per leaf	Maximum features
ACCESS1-0	rcp45	100	5	2	1	None
ACCESS1-0	rcp85	200	5	2	4	None
CESM1-CAM5	rcp45	200	5	2	4	None
CESM1-CAM5	rcp85	150	5	6	2	None
CNRM-CM5	rcp45	200	5	6	1	None
CNRM-CM5	rcp85	150	5	6	2	None
CanESM2	rcp45	100	5	6	3	None
CanESM2	rcp85	100	5	6	3	None
GFDL-ESM2M	rcp45	100	2	2	2	None
GFDL-ESM2M	rcp85	100	5	2	1	None
HadGEM2-CC	rcp45	200	5	6	1	None
HadGEM2-CC	rcp85	100	5	2	1	None
MIROC5	rcp45	200	5	2	4	None
MIROC5	rcp85	200	5	6	1	None
NorESM1-M	rcp45	200	5	2	4	None
NorESM1-M	rcp85	100	5	6	3	None

Table S2. Distribution of projected virus density values across bins, related to Figure 5
Counts and percentages of grid cells falling within predefined potential zoonotic virus density bins for each global climate model, emission scenario, and projection year (2020 and 2050). Values are aggregated across 277,084 grid cells.

year	RCP	model	[0-20]	(20-40]	(40-60]	(60-80]	(80-100]
2020	rcp45	ACCESS1-0	0	169814	100114	7156	0
2020	rcp45	CESM1-CAM5	0	171897	97799	7388	0
2020	rcp45	CNRM-CM5	0	173350	96068	7666	0
2020	rcp45	CanESM2	0	169960	98510	8614	0
2020	rcp45	GFDL-ESM2M	0	164661	112423	0	0
2020	rcp45	HadGEM2-CC	0	169763	100096	7225	0
2020	rcp45	MIROC5	0	172105	96992	7987	0
2020	rcp45	NorESM1-M	0	167472	101338	8274	0
2020	rcp85	ACCESS1-0	0	171332	98315	7437	0
2020	rcp85	CESM1-CAM5	0	169643	99483	7958	0
2020	rcp85	CNRM-CM5	0	170336	99653	7095	0
2020	rcp85	CanESM2	0	170747	98527	7810	0
2020	rcp85	GFDL-ESM2M	0	173574	96332	7178	0
2020	rcp85	HadGEM2-CC	0	169559	101011	6514	0
2020	rcp85	MIROC5	0	171414	98593	7077	0
2020	rcp85	NorESM1-M	0	166845	101896	8343	0
2050	rcp45	ACCESS1-0	0	132321	137717	7046	0
2050	rcp45	CESM1-CAM5	0	151214	121152	4718	0
2050	rcp45	CNRM-CM5	0	147075	126089	3920	0
2050	rcp45	CanESM2	0	134358	137240	5486	0
2050	rcp45	GFDL-ESM2M	0	116193	160891	0	0
2050	rcp45	HadGEM2-CC	0	131436	138259	7389	0
2050	rcp45	MIROC5	0	138850	133558	4676	0
2050	rcp45	NorESM1-M	0	130876	137955	8253	0
2050	rcp85	ACCESS1-0	0	150157	122134	4793	0
2050	rcp85	CESM1-CAM5	0	159917	112058	5109	0
2050	rcp85	CNRM-CM5	0	154888	118560	3636	0
2050	rcp85	CanESM2	0	142429	128239	6416	0
2050	rcp85	GFDL-ESM2M	0	172060	100355	4669	0
2050	rcp85	HadGEM2-CC	0	140854	129371	6859	0
2050	rcp85	MIROC5	0	139334	133198	4552	0
2050	rcp85	NorESM1-M	0	136146	133530	7408	0
			Percentage (%)				
2020	rcp45	ACCESS1-0	0	61.28610818	36.13128149	2.582610328	0
2020	rcp45	CESM1-CAM5	0	62.03786577	35.29579478	2.666339449	0
2020	rcp45	CNRM-CM5	0	62.56225549	34.67107448	2.766670035	0
2020	rcp45	CanESM2	0	61.33879979	35.55239566	3.10880455	0

year	RCP	model	[0-20]	(20-40]	(40-60]	(60-80]	(80-100]
2020	rcp45	GFDL-ESM2M	0	59.42638334	40.57361666	0	0
2020	rcp45	HadGEM2-CC	0	61.26770221	36.12478526	2.607512523	0
2020	rcp45	MIROC5	0	62.11293326	35.00454736	2.88251938	0
2020	rcp45	NorESM1-M	0	60.44087713	36.57302479	2.986098079	0
2020	rcp85	ACCESS1-0	0	61.83395649	35.48201989	2.684023617	0
2020	rcp85	CESM1-CAM5	0	61.22439405	35.90355271	2.87205324	0
2020	rcp85	CNRM-CM5	0	61.47449871	35.96490595	2.560595343	0
2020	rcp85	CanESM2	0	61.62282918	35.55853099	2.818639835	0
2020	rcp85	GFDL-ESM2M	0	62.6430974	34.76635244	2.590550158	0
2020	rcp85	HadGEM2-CC	0	61.19407833	36.45501003	2.350911637	0
2020	rcp85	MIROC5	0	61.8635504	35.58235048	2.554099118	0
2020	rcp85	NorESM1-M	0	60.21459196	36.77440776	3.011000274	0
2050	rcp45	ACCESS1-0	0	47.75483247	49.70225636	2.542911175	0
2050	rcp45	CESM1-CAM5	0	54.57334238	43.72392487	1.702732745	0
2050	rcp45	CNRM-CM5	0	53.07957154	45.50569502	1.414733438	0
2050	rcp45	CanESM2	0	48.4899886	49.53010639	1.979905011	0
2050	rcp45	GFDL-ESM2M	0	41.9342149	58.0657851	0	0
2050	rcp45	HadGEM2-CC	0	47.43543474	49.89786491	2.666700351	0
2050	rcp45	MIROC5	0	50.11115763	48.20126749	1.687574887	0
2050	rcp45	NorESM1-M	0	47.23332996	49.78815089	2.978519149	0
2050	rcp85	ACCESS1-0	0	54.19186961	44.07833004	1.729800349	0
2050	rcp85	CESM1-CAM5	0	57.71426715	40.44188766	1.843845188	0
2050	rcp85	CNRM-CM5	0	55.89929408	42.78846848	1.312237444	0
2050	rcp85	CanESM2	0	51.40282369	46.28163301	2.315543301	0
2050	rcp85	GFDL-ESM2M	0	62.0966927	36.21825872	1.685048577	0
2050	rcp85	HadGEM2-CC	0	50.83440401	46.69017338	2.475422616	0
2050	rcp85	MIROC5	0	50.2858339	48.07134299	1.642823115	0
2050	rcp85	NorESM1-M	0	49.13528028	48.19116225	2.673557477	0

SUPPLEMENTAL REFERENCES

S1. Golchin, M., Di Marco, M., Horwood, P.F., Paini, D.R., Hoskins, A.J., and Hickson, R.I. (2024). Prediction of viral spillover risk based on the mass action principle. One Health *18*, 100737. https://doi.org/10.1016/j.onehlt.2024.100737.
